# Association between 1400 blood metabolites and the risk of ankylosing spondylitis: A 2-stage, 2-sample Mendelian randomization study

**DOI:** 10.1097/MD.0000000000047598

**Published:** 2026-02-06

**Authors:** Fei Li, Xinhua Zhou, Chunning Li, Zhenfeng Zhang, Qiqi Yang

**Affiliations:** a Department of Rehabilitation, Second Affiliated Hospital of Anhui University of Chinese Medicine, Hefei, China; b Intelligent Manufacturing Institute, Hefei University of Technology, Hefei, China.

**Keywords:** 2, -deoxyuridine, ankylosing spondylitis, causality, Mendelian randomization, metabolites, phenome-wide Mendelian randomization

## Abstract

Human blood metabolites have been closely linked to ankylosing spondylitis (AS) in observational studies, yet direct causal evidence remains limited. This study aims to use Mendelian randomization (MR) to pinpoint causal metabolites associated with AS and to predict potential side effects of metabolite interventions. Genetic instruments for exposure were sourced from a genome-wide association study of 1400 blood metabolites, while genome-wide association study data for AS outcomes were derived from the FinnGen cohort. The primary MR analysis was conducted using the inverse variance weighted method. Supplemental analyses were conducted using weighted median, MR-Egger, simple mode, and weighted mode methods, while sensitivity analyses were performed to evaluate heterogeneity and pleiotropy. A replication analysis using an additional the UK Biobank cohort was also performed to determine metabolites associated with AS. The Steiger test and linkage disequilibrium score regression were used to further strengthen causal inference. Lastly, a phenome-wide Mendelian randomization analysis was performed to investigate the potential on-target side effects of metabolite interventions. After comprehensive analyses, 3 metabolites (the 2′-deoxyuridine levels, the hate to mannose ratio, and the Uridine to 2′-deoxyuridine ratio) were identified as being genetically associated with AS. The phenome-wide Mendelian randomization analysis revealed that the hate to mannose ratio might have deleterious effects on 4 other diseases, while no significant associations were found for the 2′-deoxyuridine levels or the uridine to 2′-deoxyuridine ratio with other diseases. This systematic MR analysis unveiled the potential role of the 2′-deoxyuridine levels, hate to mannose ratio and uridine to 2′-deoxyuridine ratio as the causal mediator in the development of AS. Considering the advantages and disadvantages, 2′-deoxyuridine appears as the most promising prospective therapeutic target for the prevention of AS.

## 1. Introduction

Ankylosing spondylitis (AS) is a chronic, progressive inflammatory joint disease predominantly affecting the spinal and sacroiliac joints, and is a subtype of axial spondylitis.^[1,2]^ Clinically, AS is characterized by severe pain, restricted spinal movement, and abnormalities in spinal mobility, which can progress to joint deformity and irreversible disability in advanced stages.^[3]^ Epidemiological research indicates that AS has a worldwide incidence rate ranging from 0.1% to 1.4%, with a 2- to 3-fold higher in incidence in males than females.^[4,5]^ There is no doubt that AS has imposed a significant burden on both patients and healthcare systems worldwide as a whole.

One of the critical challenges in managing AS is the difficulty in early diagnosis.^[6]^ Early-stage AS often presents asymptomatically or with nonspecific back pain, leading to delays in diagnosis that average between 5 and 10 years.^[6,7]^ Such delays not only exacerbate the disease’s impact on AS patient mobility and quality of life but also significantly increase treatment costs.^[8]^ Although HLA-B27 is recognized as an important diagnostic marker for AS, its low specificity and high prevalence in the general population limit its utility as a definitive diagnostic tool.^[9]^ Similarly, traditional markers like erythrocyte sedimentation rate and C-reactive protein levels are commonly used to monitor disease progression, but their low sensitivity and specificity limit them to serving merely as reference indicators.^[10,11]^ Novel biomarkers that can facilitate early diagnosis of AS and improved patient outcomes are thus desperately needed.

Metabolites, as the end products of biochemical processes, are crucial indicators reflecting the physiological and pathological states of the body.^[12]^ Numerous metabolomics studies have been carried out to characterize the metabolic profiles of AS patients,^[13,14]^ with findings revealing perturbations in key metabolites such as tryptophan, lysine, proline, serine, and alanine.^[15-18]^ These metabolic disturbances offer significant clues for exploring the pathogenesis of AS. However, while these studies have identified associations between certain metabolites and AS in population-based cohorts, the inherent limitations of conventional observational research-such as susceptibility to confounding and the inability to infer causality-prevent current evidence from definitively establishing a metabolic spectrum that contributes to AS development. This gap underscores the need for more robust analytical approaches to accurately delineate the causal roles of these metabolites in the onset and progression of AS.

Mendelian randomization (MR) is a robust technique that leverages genetic variants as proxies to estimate the causality effects of specific exposures on disease risk, effectively overcomes the shortcomings of conventional observational research.^[19,20]^ While MR has been utilized to evaluate the influence of various biomarkers, such as inflammatory regulators and serum DKK-1, on AS risk, a comprehensive exploration of the human metabolome to identify causal mediators of AS remains absent.^[21,22]^ This study aims to address this gap by systematically investigating 1400 circulating metabolites using MR to identify promising mediators of AS. Moreover, the phenome-wide Mendelian randomization (Phe-MR) analysis is capable of identifying possible side effects of prospective drug targets before they enter clinical trials.^[23]^ To this end, we conducted a Phe-MR analysis across 784 disease traits to anticipate potential side effects associated with metabolite-targeted interventions, ensuring a comprehensive evaluation of their clinical safety.

## 2. Material and methods

### 2.1. Study design

**Figure 1.**
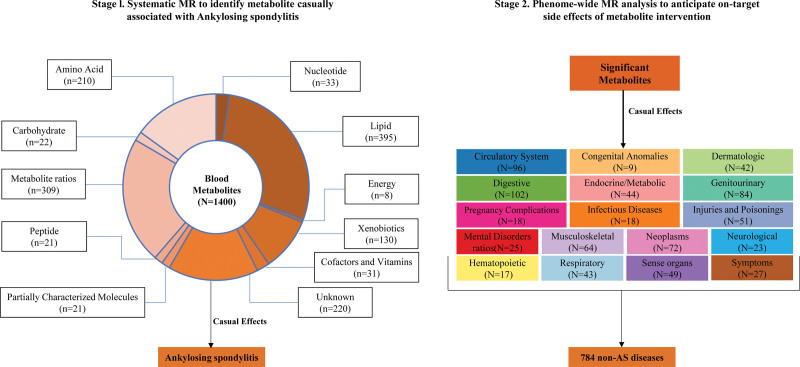
Conceptual framework of 2-stage MR study. This study follows a 2-stage design, incorporating MR analysis at each stage. In the first stage, we evaluated the causal relationships between 1400 blood metabolites and the risk of AS. In the second stage, we explored the potential side effects associated with targeting the identified metabolites across 784 non-AS diseases. AS = ankylosing spondylitis, MR = Mendelian randomization.

This study adhered to the Strengthening the Reporting of Observational Studies in Epidemiology using Mendelian Randomization (STROBE-MR) guidelines.^[24]^ As shown in Figure 1, a 2-stage MR approach was employed to systematically determine potential causal mediators for AS along with their associated target-mediated side effects. MR method relies on 3 core assumptions: genetic variants directly influence the exposures, these variants are independent of potential confounders, and their effect on outcomes is mediated solely through the exposures.^[25]^ Aggregated data for blood metabolome, AS, and 784 other non-AS diseases were sourced from publicly available genome-wide association study (GWAS) of European descent populations. The original GWAS received ethics approval and obtained informed consent from participants, exempting this study from additional ethics review.

All statistical analyses were carried out using R software (v4.2.1), with the aid of several packages including “TwoSampleMR”(v0.5.4), “MendelianRandomization” (v0.9.0), “ieugwasr” (v0.1.5), “forestplot” (v1.1.1), “ldscr” (v0.1.0),“ComplexHeatmap” (v2.20.0), “ggplot2” (v3.4.3).

### 2.2. GWAS data for blood metabolites

Metabolite data were derived from 8299 participants within the Canadian longitudinal study of aging cohort, which is the most comprehensive and the latest dataset for blood metabolites.^[26]^ The data included 1091 metabolites and 309 metabolite ratios, were specifically derived from a large-scale genome-wide association scan and high-throughput metabolic analysis carried out by Chen et al. Of these 1091 plasma metabolites, 850 were labeled as 8 major super pathways (amino acids, carbohydrate, cofactors and vitamins, energy, lipid, nucleotide, peptide, and xenobiotics). The remaining 241 metabolites were identified as either unknown or partially characterized molecules. Genetic information of 1400 blood metabolites can be available in the GWAS Catalog database (https://www.ebi.ac.uk/gwas) under catalog numbers GCST90199621-GCS90201020 (Table S1, Supplemental Digital Content, https://links.lww.com/MD/R339).

### 2.3. GWAS data for AS

Genetic data for AS were derived from the FinnGen consortium (https://r11.finngen.fi/), involving a study cohort of individuals of European ancestry. The FinnGen initiative synthesized genetic information relevant to disease outcomes from both the Finnish Biobank and the Finnish Health Registry. This dataset included 4,51,998 individuals with 1521 cases and 4,50,477 controls. Cases were identified using codes from the Tenth Revision of the International Classification of Diseases (ICD-10). To support our findings using duplicate analysis, we used data from the UK Biobank (UKB) on AS, which contained 3,37,159 European individuals, 968 cases, and 3,36,191 controls. The data is publically available through the IEU OpenGWAS project (https://gwas.mrcieu.ac.uk/) with the accession number ukb-a-88.

### 2.4. Instruments selection

A series of systematic approaches was undertaken to screen instrumental variables (IVs) correlated with blood metabolites. We imposed a correlation threshold of 1 × 10^−5^ (pairwise linkage disequilibrium *r*^2^ < 0.1 within a 500 kb distance) to identify single nucleotide polymorphism (SNPs) significantly linked to metabolites.^[27-29]^ *F*-statistics were computed for each IV, and those with an *F*-value below 10 were considered weak and eliminated from subsequent analysis. We then extracted SNPs associated with the exposure from the outcome data, removing any SNPs that were also associated with the outcome at a significance level of *P* < 1 × 10^–5^. For SNPs missing in the outcome dataset, high linkage disequilibrium proxies (*r*^2^ > 0.8) were identified using the European reference panel from the 1000 Genomes Project, and SNPs without suitable proxies were excluded. Next, we performed harmonization to ensure consistent alignment of alleles between exposure and outcome SNPs. This process involved the exclusion of palindromic SNPs with high effect allele frequencies (EAF > 0.42) or with ambiguous alleles (e.g., A/G vs A/C). Additionally, certain MR sensitivity analyses required a minimum of 3 SNPs associated with the exposure to serve as the genetic instrument, leading to the exclusion of metabolites that were associated with fewer than 3 SNPs.^[30]^

### 2.5. Primary analysis and sensitivity analysis

For the analysis of metabolite-AS associations, we applied 5 MR methods for analysis (inverse variance weighted [IVW], weighted median [WM], MR-Egger, weighted mode, and simple mode). IVW was designated as the primary statistical approach. Considering the exploratory nature of this MR study and the potential for the Bonferroni correction to miss out metabolites involved in AS development, we set a threshold of *P* < .05 for IVW analysis to identify suggestive significant associations, this threshold extensively used in previous studies.^[31,32]^ The WM, MR-Egger, weighted mode, and simple mode served as supplementary methods. A metabolite was deemed a candidate if the estimates across all 5 models were consistent in both direction and magnitude. Meanwhile, we also performed the Cochran Q test to evaluate heterogeneity, which was considered significant if the test yielded *P* < .05 and *I*^2^ > 25%.^[33]^ The Egger intercept was used to assess horizontal pleiotropy.^[25]^ At last, leave-one-out (LOO) analysis was performed to determine the impact of individual SNPs on the primary causal estimates.

To sum up, the potential eligible candidate metabolites associated with AS were identified based on the following criteria: IVW derived *P* < .05; consistent directions and magnitudes across 5 MR methods; absence of heterogeneity and pleiotropy; and no significant influence from any single SNP as revealed by LOO analysis.

### 2.6. Replication analysis

To validate the reliability of candidate metabolites, we executed a replication of the IVW analysis utilizing an independent AS GWAS dataset from the UKB consortium. Only those metabolites that showed consistent associations in both the FinnGen-MR and UKB-MR were recognized as the final candidates with genetic links to AS.

### 2.7. Genetic correlation and direction validation

Despite MR estimates are valuable, the estimates may diverge from the true effects when there is a genetic linkage between exposure and outcome.^[34]^ In our analysis, we excluded SNPs specifically related to AS when selecting IVs; however, SNPs that are not directly linked to AS could still influence its genetic architecture. To resolve potential violations arising from genetic correlations, we utilized linkage disequilibrium score (LDSC) regression, which evaluates coinheritance by analyzing Chi-squared statistics derived from SNPs associated with 2 traits. Additionally, the Steiger test was applied to mitigate potential bias caused by reverse associations.^[35]^

### 2.8. Phe-MR analysis of 784 phenotypes for identified AS-associated metabolites

The Phe-MR analysis was carried out to evaluate the potential adverse effects of targeting AS-associated metabolites on the risk of other diseases. Summary data for 1403 disease traits were sourced from the GWAS by Zhou et al, which involved 4,08,961 white British participants and examined 28 million SNPs in the UKB cohort.^[36]^ Disease traits were classified using the “Phecodes” system, which was designed to convert International Classification of Diseases codes into phenotypic outcomes suitable for comprehensive genetic analysis across multiple traits.^[37]^ Disease traits with fewer than 500 cases were excluded from the analysis due to limited data availability and statistical power.^[38]^ Ultimately, 784 diseases were included in this Phe-MR analysis (Table S2, Supplemental Digital Content, Supplemental Digital Content, https://links.lww.com/MD/R339).

The Phe-MR results revealed the risk or protective effect corresponding to each standard deviation increase in blood metabolite levels. In our analysis, a metabolite was considered as having potential beneficial effects on other diseases when used as a therapeutic target for AS if the metabolite-AS association was in the same direction as the metabolite-other diseases associations.^[39]^ Otherwise, it might have deleterious effects for the other diseases. For Phe-MR causal effects, Bonferroni-adjusted α-levels were considered statistically significant as 2.13 × 10^−5^ (0.05/[3 × 784]), for 3 metabolites and 784 phenotypes.

## 3. Results

### 3.1. IVs for exposures

Following the instrument selection process, 1400 metabolites were incorporated into the MR analysis. A total of 37,108 SNPs were identified, with the number of SNPs per metabolite ranging from 11 to 104. All selected SNPs exhibited *F* statistics >10, indicating a lack of weak instruments. Detailed information on IVs is available in Table S3, Supplemental Digital Content, https://links.lww.com/MD/R339.

### 3.2. Association of blood metabolites with AS

**Figure 2.**
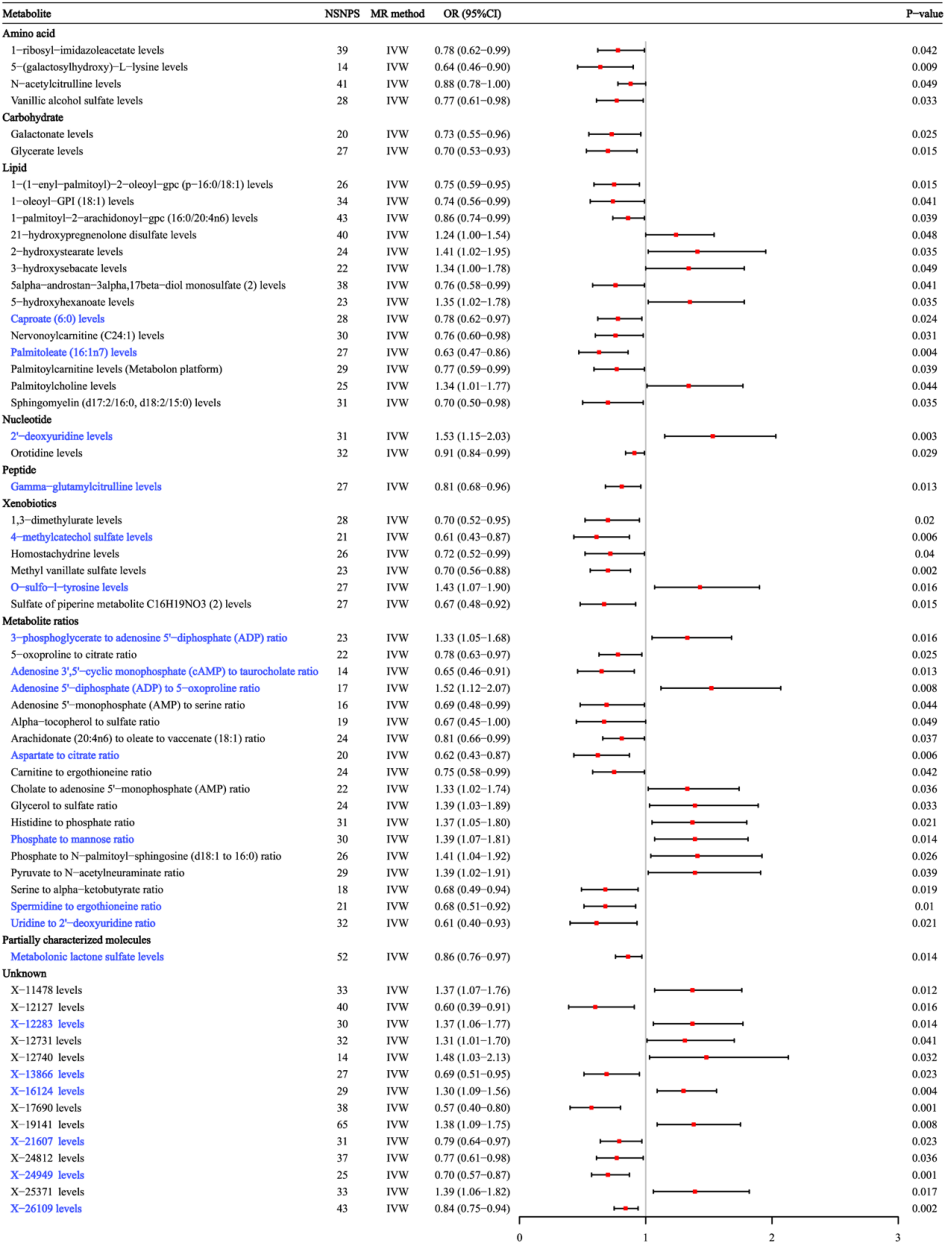
Forest plot for 62 blood metabolites with potential causal associations with AS identified by IVW method. AS = ankylosing spondylitis, CI = confidence interval, IVW = inverse variance weighted, MR = Mendelian randomization, NSNPS = number of single nucleotide polymorphisms, OR = odds ratio.

The initial IVW analysis indicated a potential correlation between 62 metabolites and AS (*P* < .05 for IVW), including 40 protective and 22 risk variables (Fig. 2, Table S4, Supplemental Digital Content, https://links.lww.com/MD/R340). Fourty eight of them were identified and classified as amino acid, carbohydrate, lipid, nucleotide, peptide, xenobiotics, partially characterized molecules, and metabolite ratios, while 14 were chemically unknown. Subsequent supplementary analyses and sensitivity analyses reduced these metabolites to 20 candidates potentially involved in AS development (Table 1). Out of the 20 metabolites, 13 were potentially associated with a decreased risk of AS: caproate (6:0) levels (OR = 0.78, 95% CI: 0.62–0.97, *P* = .024), palmitoleate (16:1n7) levels (OR = 0.63, 95% CI: 0.47–0.86, *P* = .004), γ-glutamylcitrulline levels (OR = 0.81, 95% CI: 0.68–0.96, *P* = .013), 4-methylcatechol sulfate levels (OR = 0.61, 95% CI: 0.43–0.87, *P* = .006), adenosine 3′,5′-cyclic monohate (cAMP) to taurocholate ratio (OR = 0.65, 95% CI: 0.46–0.91, *P* = .013), aspartate to citrate ratio (OR = 0.62, 95% CI: 0.43–0.87, *P* = .006), spermidine to ergothioneine ratio (OR = 0.68, 95% CI: 0.51–0.92, *P* = .01), uridine to 2′-deoxyuridine ratio (OR = 0.61, 95% CI: 0.40–0.93, *P* = .021), metabolonic lactone sulfate levels (OR = 0.86, 95% CI: 0.76–0.97, *P* = .014), X-13866 levels (OR = 0.69, 95% CI: 0.51–0.95, *P* = .023), X-21607 levels (OR = 0.79, 95% CI: 0.64–0.97, *P* = .023), X-24949 levels (OR = 0.70, 95% CI: 0.57–0.87, *P* = .001), and X-26109 levels (OR = 0.84, 95% CI: 0.75–0.94, *P* = .002). On the other hand, 7 metabolites were potentially associated with an increased risk of AS: 2′-deoxyuridine levels (OR = 1.53, 95% CI: 1.15–2.03, *P* = .003), *O*-sulfo-l-tyrosine levels (OR = 1.43, 95% CI: 1.07–1.90, *P* = .016), 3-hoglycerate to adenosine 5′-dihate (ADP) ratio (OR = 1.33, 95% CI: 1.05–1.68, *P* = .016), adenosine 5′-dihate (ADP) to 5-oxoproline ratio (OR = 1.52, 95% CI: 1.12–2.07, *P* = .014), hate to mannose ratio (OR = 1.39, 95% CI: 1.07–1.81, *P* = .008), X-12283 levels (OR = 1.37, 95% CI: 1.06–1.77, *P* = .014), and X-16124 levels (OR = 1.30, 95% CI: 1.09–1.56, *P* =* *.004).

**Table 1:** Complementary and sensitivity analyses for genetic association between 20 blood metabolites and AS.

Metabolites	N	MR analysis			Heterogeneity		Pleiotropy	
		Method	OR (95% CI)	*P*-value	*Q*	*P*-value	Intercept	*P*-value
Lipid								
Caproate (6:0) levels	28	IVW	0.78 (0.62–0.97)	.024	28.433	.389	0.033	.493
		WM	0.74 (0.53–1.02)	.066				
		MR-Egger	0.69 (0.46–1.03)	.081				
		Simple mode	0.63 (0.36–1.13)	.132				
		Weighted mode	0.74 (0.51–1.06)	.108				
Palmitoleate (16:1n7) levels	27	IVW	0.63 (0.47–0.86)	.004	20.684	.758	0.059	.186
		WM	0.68 (0.44–1.07)	.094				
		MR-Egger	0.39 (0.18–0.84)	.023				
		Simple mode	0.63 (0.38–1.05)	.090				
		Weighted mode	0.74 (0.51–1.06)	.110				
Nucleotide								
2′-Deoxyuridine levels	31	IVW	1.53 (1.15–2.03)	.003	41.953	.072	0.043	.287
		WM	1.41 (0.97–2.06)	.074				
		MR-Egger	1.14 (0.63–2.08)	.667				
		Simple mode	1.08 (0.56–2.06)	.822				
		Weighted mode	1.24 (0.84–1.85)	.291				
Peptide								
λ-glutamylcitrulline levels	27	IVW	0.81 (0.68–0.96)	.013	14.516	.966	−0.034	.162
		WM	0.84 (0.66–1.07)	.152				
		MR-Egger	0.90 (0.72–1.13)	.381				
		Simple mode	0.66 (0.38–1.14)	.146				
		Weighted mode	0.83 (0.67–1.04)	.123				
Xenobiotics								
4-Methylcatechol sulfate levels	21	IVW	0.61 (0.43–0.87)	.006	20.155	.448	−0.012	.796
		WM	0.68 (0.42–1.09)	.112				
		MR-Egger	0.68 (0.28–1.68)	.415				
		Simple mode	0.56 (0.24–1.32)	.201				
		Weighted mode	0.60 (0.28–1.29)	.206				
*O*-sulfo-l-tyrosine levels	27	IVW	1.43 (1.07–1.90)	.016	28.525	.333	−0.023	.576
		WM	1.55 (1.05–2.29)	.029				
		MR-Egger	1.68 (0.89–3.19)	.124				
		Simple mode	1.43 (0.76–2.67)	.280				
		Weighted mode	1.50 (0.87–2.58)	.154				
Metabolite ratios								
3-Phosphoglycerate to adenosine 5′-dihate (ADP) ratio	23	IVW	1.33 (1.05–1.68)	.016	14.792	.871	−0.022	.630
		WM	1.29 (0.92–1.82)	.146				
		MR-Egger	1.48 (0.92–2.36)	.120				
		Simple mode	1.54 (0.84–2.80)	.174				
		Weighted mode	1.33 (0.84–2.11)	.240				
Adenosine 3′,5′-cyclic monophosphate (cAMP) to taurocholate ratio	14	IVW	0.65 (0.46–0.91)	.013	7.342	.884	0.031	.569
		WM	0.69 (0.44–1.08)	.103				
		MR-Egger	0.53 (0.24–1.14)	.130				
		Simple mode	0.89 (0.39–2.04)	.784				
		Weighted mode	0.86 (0.40–1.88)	.718				
Adenosine 5′-diphosphate (ADP) to 5-oxoproline ratio	17	IVW	1.52 (1.12–2.07)	.008	18.620	.289	−0.072	.251
		WM	1.42 (0.96–2.11)	.076				
		MR-Egger	2.23 (1.11–4.47)	.040				
		Simple mode	1.94 (1.02–3.71)	.061				
		Weighted mode	1.75 (1.00–3.07)	.066				
Aspartate to citrate ratio	20	IVW	0.62 (0.43–0.87)	.006	13.927	.788	0.031	.600
		WM	0.78 (0.49–1.25)	.301				
		MR-Egger	0.50 (0.21–1.18)	.131				
		Simple mode	0.69 (0.32–1.45)	.338				
		Weighted mode	0.81 (0.47–1.43)	.482				
Phosphate to mannose ratio	30	IVW	1.39 (1.07–1.81)	.014	32.696	.290	0.018	.681
		WM	1.44 (0.99–2.11)	.059				
		MR-Egger	1.26 (0.74–2.15)	.399				
		Simple mode	1.56 (0.76–3.21)	.234				
		Weighted mode	1.60 (1.07–2.39)	.030				
Spermidine to ergothioneine ratio	21	IVW	0.68 (0.51–0.92)	.010	22.744	.302	−0.023	.593
		WM	0.69 (0.47–1.02)	.060				
		MR-Egger	0.81 (0.42–1.55)	.525				
		Simple mode	0.80 (0.41–1.55)	.516				
		Weighted mode	0.69 (0.41–1.17)	.185				
Uridine to 2′-deoxyuridine ratio	32	IVW	0.61 (0.40–0.93)	.021	27.830	.375	−0.033	.621
		WM	0.68 (0.48–0.96)	.028				
		MR-Egger	0.79 (0.27–2.36)	.679				
		Simple mode	0.90 (0.47–1.70)	.742				
		Weighted mode	0.62 (0.36–1.08)	.099				
Partially Characterized Molecules								
Metabolonic lactone sulfate levels	52	IVW	0.86 (0.76–0.97)	.014	57.119	.258	0.004	.865
		WM	0.88 (0.74–1.04)	.142				
		MR-Egger	0.85 (0.71–1.01)	.074				
		Simple mode	0.82 (0.57–1.18)	.288				
		Weighted mode	0.91 (0.78–1.06)	.218				
Unknown								
X-12283 levels	30	IVW	1.37 (1.06–1.77)	.014	32.195	.311	0.007	.845
		WM	1.30 (0.86–1.98)	.213				
		MR-Egger	1.31 (0.77–2.22)	.324				
		Simple mode	2.47 (1.16–5.26)	.027				
		Weighted mode	1.35 (0.79–2.30)	.281				
X-13866 levels	27	IVW	0.69 (0.51–0.95)	.023	27.969	.360	−0.029	.514
		WM	0.87 (0.56–1.36)	.533				
		MR-Egger	0.88 (0.41–1.89)	.742				
		Simple mode	0.87 (0.42–1.79)	.712				
		Weighted mode	0.94 (0.52–1.71)	.837				
X-16124 levels	29	IVW	1.30 (1.09–1.56)	.004	23.718	.696	0.033	.254
		WM	1.15 (0.88–1.49)	.306				
		MR-Egger	1.17 (0.91–1.51)	.228				
		Simple mode	1.27 (0.79–2.06)	.333				
		Weighted mode	1.18 (0.92–1.51)	.196				
X-21607 levels	31	IVW	0.79 (0.64–0.97)	.023	31.829	.376	0.015	.694
		WM	0.97 (0.72–1.32)	.864				
		MR-Egger	0.73 (0.48–1.11)	.154				
		Simple mode	0.39 (0.21–0.72)	.006				
		Weighted mode	0.97 (0.74–1.29)	.860				
X-24949 levels	25	IVW	0.70 (0.57–0.87)	.001	20.740	.654	−0.033	.329
		WM	0.73 (0.52–1.04)	.078				
		MR-Egger	0.84 (0.56–1.27)	.423				
		Simple mode	0.67 (0.39–1.14)	.155				
		Weighted mode	0.75 (0.51–1.10)	.153				
X-26109 levels	43	IVW	0.84 (0.75–0.94)	.002	33.432	.825	0.029	.180
		WM	0.88 (0.74–1.05)	.163				
		MR-Egger	0.78 (0.66–0.91)	.003				
		Simple mode	0.77 (0.56–1.04)	.099				
		Weighted mode	0.86 (0.74–1.01)	.067				

ADP = adenosine 5'-dihate, AS = ankylosing spondylitis, CI = confidence interval, IVW = inverse variance weighted, MR = Mendelian randomization, OR = odds ratio, WM = weighted median.

**Figure 3.**
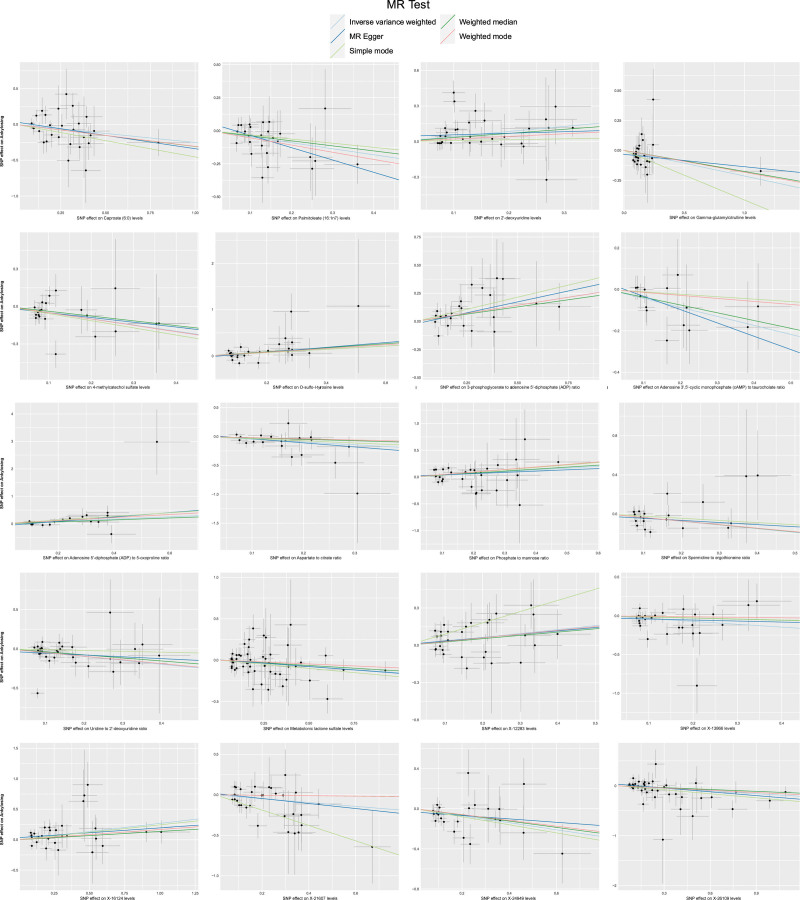
Scatterplot for 20 blood metabolites with significant associations with AS identified by complementary and sensitivity analyses. AS = ankylosing spondylitis, SNP = single nucleotide polymorphism.

In summary, the IVW-derived estimates were statistically significant (*P < *.05), and the estimates from the IVW, WM, MR-Egger, weighted mode, and simple mode were consistent in both direction and magnitude (Fig. 3). Both the Cochran *Q* test and the MR-Egger intercept test provided strong evidence against heterogeneity and pleiotropy (*P < *.05; Table 1). LOO analysis confirmed that MR estimation was not biased by any single SNP (Fig. S1, Supplemental Digital Content, https://links.lww.com/MD/R341). The funnel plots were showed on Figure S2, Supplemental Digital Content, https://links.lww.com/MD/R341. Consequently, these 20 blood metabolites have been identified as strong candidates for further replication analysis.

### 3.3. Replication analysis

**Figure 4.**
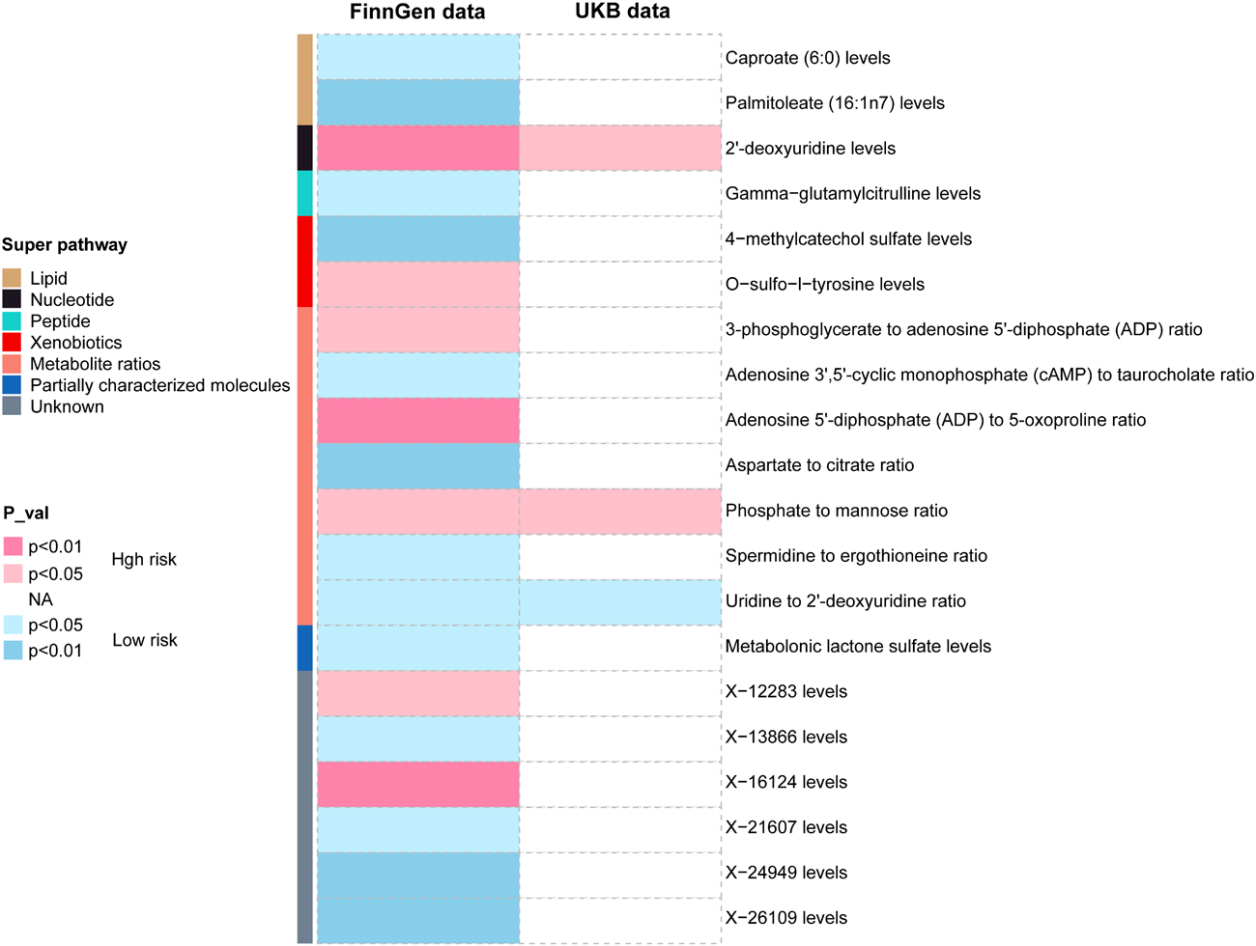
Heatmap of causal associations between blood metabolites and AS derived from IVW analysis. AS = ankylosing spondylitis, IVW = inverse variance weighted.

To ascertain the reliability of candidate metabolites, we performed a duplicate IVW analysis using a separate AS GWAS dataset obtained from the previously stated UKB consortium. After the replication analysis, only 3 metabolites which were consistent with FinnGen-MR, including 2′-deoxyuridine levels, hate to mannose ratio, and uridine to 2′-deoxyuridine ratio. They were considered to be the final candidates with genetic associations on AS (Fig. 4).

### 3.4. Genetic correlation and direction validation

LDSC analysis demonstrated an insignificant genetic link between AS and 2′-deoxyuridine levels (Rg = 0.2370, se = 0.6459, *P* = .7137), hate to mannose ratio (Rg = 0.3552, se = 0.2823, *P* = .2083), and Uridine to 2′-deoxyuridine ratio (Rg = 0.0593, se = 0.3855, *P* = .8777). This suggests that confounding shared genetic components are absent in the MR estimates. Furthermore, the Steiger test results indicated no evidence of reverse causality between the metabolites and AS (Table 2).

**Table 2:** Steiger direction test from blood metabolites to AS.

Exposure	2′-Deoxyuridine levels	Phosphate to mannose ratio	Uridine to 2′-deoxyuridine ratio
Direction	True	True	True
Steiger *P*	1.70E−217	1.51E−176	1.53E−154

AS = ankylosing spondylitis.

### 3.5. Phe-MR analysis for the associations between identified metabolites and 784 diseases

**Figure 5.**
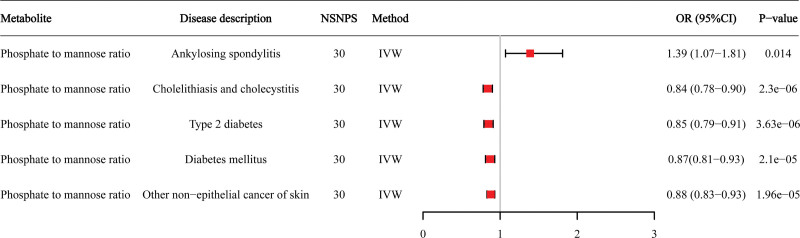
Forest plot of Phe-MR analysis for causal associations between hate to mannose ratio and risk of AS and multiple non-AS diseases. AS = ankylosing spondylitis, CI = confidence interval, IVW = inverse variance weighted, NSNPS = number of single nucleotide polymorphisms, OR = odds ratio, Phe-MR =  phenome-wide Mendelian randomization.

We conducted a Phe-MR analysis to investigate whether AS-related metabolites were linked to 784 other diseases (Tables S5–S7, Supplemental Digital Content, https://links.lww.com/MD/R340). Four associations between 3 metabolites and non-AS diseases met the Bonferroni-corrected significance threshold of *P* < 2.13 × 10^−5^ (Table 3). In this Phe-MR analysis, a metabolite was deemed potentially beneficial for other diseases when its associations with both AS and those diseases were in the same direction; otherwise, it might have harmful effects. To be specific, hate to mannose ratio showed likely deleterious effects on cholelithiasis and cholecystitis, type 2 diabetes, other non-epithelial cancer of skin and diabetes mellitus. These results suggest that targeting the hate to mannose ratio for AS treatment might have potential adverse reactions. Additionally, no significant beneficial or deleterious effects were observed between 2′-deoxyuridine levels or the uridine to 2′-deoxyuridine ratio and 784 common non-AS-related diseases (Fig. 5).

**Table 3:** Phe-MR analyses for causal associations of the hate to mannose ratio with the risk of multiple non-AS diseases.

PheCode	Phenotype description	Phenotype category	MR method	NSNP	OR (95% CI)	*P*-value
574	Cholelithiasis and cholecystitis	Digestive	IVW	30	0.85 (0.78–0.90)	2.30E−06
250.2	Type 2 diabetes	Endocrine/metabolic	IVW	30	0.85(0.79–0.91)	3.63E−06
172.2	Other non-epithelial cancer of skin	Neoplasms	IVW	30	0.88 (0.83–0.93)	1.96E−05
250	Diabetes mellitus	Endocrine/metabolic	IVW	30	0.87 (0.81–0.93)	2.10E−05

AS = ankylosing spondylitis, CI = confidence interval, IVW = inverse variance weighted, MR = Mendelian randomization, NSNPS = number of single nucleotide polymorphisms, OR = odds ratio, Phe-MR = phenome-wide Mendelian randomization.

## 4. Discussion

To our knowledge, this is the first study to use a 2-stage, 2-sample MR analysis to investigate the causative impact of 1400 blood metabolites on AS risk. We identified 3 key metabolites associated with AS, highlighting their crucial role in its pathogenesis. Specifically, elevated levels of 2′-deoxyuridine and a higher hate-to-mannose ratio were found to increase AS risk, while a higher uridine-to-2′-deoxyuridine ratio was associated with a reduced risk. Our study also utilized Phe-MR analysis to predict the on-target side effects of potential treatments targeting these metabolites. We found that a higher hate-to-mannose ratio was associated with increased risks of cholelithiasis, cholecystitis, type 2 diabetes, non-epithelial skin cancer and diabetes mellitus, while the 2′-deoxyuridine levels and the uridine to 2′-deoxyuridine ratio showed no potential association with the risk of other non-AS disease.

Currently, the treatment for AS remains a significant clinical challenge within the medical field.^[40]^ Conventional diagnostic methods for AS rely on clinical symptoms and imaging examinations; however, these methods exhibit a low sensitivity during the early stages of the disease and are inadequate for predicting disease progression, thereby severely impeding the effective diagnosis and management of AS.^[41,42]^ Recent studies have increasingly identified notable alterations in certain blood metabolites among AS patients.^[43-45]^ These metabolites are not only easy to measure, but also reflect the body’s inflammatory state and bone metabolism changes, and possess the capability to serve as therapeutic intervention targets. Nevertheless, the ambiguous relationship between these abnormalities and AS limits their utility in early detection. Our MR study aims to elucidate this relationship, potentially guiding future AS screening and treatment strategies.

In this study, we observed that the 2′-deoxyuridine level is associated with an increased risk of AS. Although research on the link between 2′-deoxyuridine and AS is limited, some indirect evidence and theoretical assumptions suggest that 2′-deoxyuridine may affect AS risk by influencing inflammatory responses and cellular stress. As a deoxyribonucleoside that integrates into DNA, 2′-deoxyuridine can cause DNA damage and activate DNA repair mechanisms.^[46,47]^ This damage can trigger cellular stress responses, subsequently resulting in the generation of reactive oxygen species and cytokines such as hydrogen peroxide (H_2_O_2_), superoxide (O_2_^−^), tumor necrosis factor-alpha (TNF-α), and interleukin-6 (IL-6).^[48]^ These signaling molecules can activate the immune system and provoke inflammatory responses.^[49]^ Furthermore, studies have shown that DNA damage and repair activation can lead to inflammatory disorders such as AS, RA, and systemic lupus erythematosus.^[50-54]^ Given that chronic inflammation and immune abnormalities are key features of AS, we postulate that DNA damage caused by 2′-deoxyuridine may contribute to its genesis and progression by activating cellular stress and inflammatory responses.

Notably, the findings pertaining to the uridine to 2′-deoxyuridine ratio further substantiates the link between increased levels of 2′-deoxyuridine and an increased risk of AS. Our MR analysis indicates that the ratio of uridine to 2′-deoxyuridine is inversely related to the risk of AS. It is widely recognized that the results for ratios may be influenced by the individual components (uridine or 2′-deoxyuridine) of that ratio.^[55]^ However, our analysis demonstrates that the uridine levels are not associated with AS (*P* > .05), whereas higher levels of 2′-deoxyuridine are associated with an elevated risk. It can be seen that the correlation between the uridine to 2′-deoxyuridine ratio and AS risk is likely driven by 2′-deoxyuridine levels. The significance of this ratio lies in its ability to support the pathological role of 2′-deoxyuridine in AS from an alternative perspective. Furthermore, our Phe-MR analysis expands our understanding of the potential side effects associated with reducing 2′-deoxyuridine levels or increasing the uridine to 2′-deoxyuridine ratio to prevent AS. The Phe-MR analysis reveals no significant associations between the 2′-deoxyuridine levels or uridine to 2′-deoxyuridine ratio and 784 non-AS diseases, thereby affirming the feasibility and safety of targeting 2′-deoxyuridine as a therapeutic strategy for AS prevention. In summary, our study substantiates that 2′-deoxyuridine can serve as a valuable biomarker and potential therapeutic target for AS. This finding underscores its prospective clinical significance in AS, and we recommend that future research should further investigate specific pathological mechanisms and clinical applications of 2′-deoxyuridine.

Additionally, we have also confirmed that a genetic predisposition to higher levels of the hate to mannose ratio is detrimental to AS. In this study, while the individual levels of hate and mannose are not associated with the risk of AS, their ratio is significantly related to AS risk. This suggests that evaluating each component separately may not capture their interactions and overall effects within complex metabolic networks. hate plays a critical role in energy metabolism, signal transduction, and nucleic acid synthesis, and is also associated with vascular calcification and inflammation in chronic inflammatory diseases.^[56,57]^ In patients with AS, dysregulated hate metabolism may exacerbate inflammatory responses and promote disease progression. On the other hand, mannose is essential for the synthesis of glycoproteins and glycolipids, and proper mannose metabolism is crucial for protein glycosylation and immune responses.^[58,59]^ Disruption in mannose metabolism could lead to immune dysregulation observed in AS. Therefore, the hate-to-mannose ratio, as an independent indicator, may reflect a more complex metabolic state, indicating disruptions in cellular metabolism and signaling pathways related to AS. This ratio could reveal intricate relationships and synergistic effects that are not apparent when measuring the levels of each component individually, serving as a potential marker of metabolic dysregulation to explain the pathophysiological mechanisms of AS. Furthermore, our Phe-MR analysis also suggested that the hate to mannose ratio has detrimental effects on cholelithiasis and cholecystitis, type 2 diabetes, other non-epithelial cancer of skin and diabetes mellitus. Therefore, the hate to mannose ratio may serve as a potential therapeutic target for AS, but caution about potential undesirable side effects should be applied in clinical practice.

Our study possesses several notable strengths. Firstly, we utilized the most advanced and comprehensive GWAS data, covering 1091 blood metabolites and 309 metabolite ratios, making it the most extensive and systematic MR study of metabolic profiles related with AS yet. Secondly, we conducted strict MR analyses that effectively overcame inherent limitations reported in earlier studies, such as reverse causality and confounding interference. Thirdly, we validated our findings using additional GWAS datasets with larger sample sizes, thereby enhancing the reliability of the results. Furthermore, we used LDSC to assess the heritability of IVs and the genetic association between metabolites and AS, increasing confidence in our MR estimations. Lastly, the application of Phe-MR analysis allowed us to investigate the causal impact of blood metabolites on a broad spectrum of diseases, aiding in the identification of potential drug targets and the prediction of on-target side effects.

However, several limitations must be acknowledged. Our reliance on data from European populations may limit the generalizability of our findings, and validation in more diverse populations is necessary. Additionally, although 1400 metabolites were tested in this study, we did not apply multiple testing correction to the MR results. As an exploratory study, we believe that such corrections could potentially miss out significant findings. Instead, we replicated the MR findings in another independent cohort to obtain candidate metabolites involved in AS risk, which is similar to a previous MR analysis conducted by Cai et al. Besides, diseases with low hospital admission rates may be underrepresented in the PheCode system due to its reliance on hospital diagnoses. Finally, while MR analysis provides valuable etiological insights, additional evidence triangulation is necessary to solidify our conclusions. Further randomized controlled trials and basic research are essential before these findings can be translated into clinical practice.

## 5. Conclusion

This systematic MR study identified the 2′-deoxyuridine levels, hate to mannose ratio and uridine to 2′-deoxyuridine ratio as potential causal mediators in the development of AS. Considering the advantages and disadvantages, 2′-deoxyuridine emerges as the most promising prospective therapeutic target for AS. These findings lay a foundation and provide new perspectives for future mechanistic research of AS, although further studies are needed for validation.

## Acknowledgments

We extend our heartfelt gratitude to all the participants of the FinnGen consortium, UK Biobank, and the Canadian Longitudinal Study of Aging cohort. Their invaluable contributions were essential to the completion of this study.

## Author contributions

**Conceptualization:** Fei Li, Qiqi Yang.

**Data curation:** Xinhua Zhou, Zhenfeng Zhang.

**Formal analysis:** Chunning Li, Qiqi Yang.

**Methodology:** Zhenfeng Zhang.

**Software:** Qiqi Yang.

**Supervision:** Fei Li.

**Writing – original draft:** Fei Li, Xinhua Zhou, Chunning Li, Qiqi Yang.

**Writing – review & editing:** Fei Li, Qiqi Yang.

## Supplementary Material






