# Supplementary material for: Association between 1400 blood metabolites and the risk of ankylosing spondylitis: A 2-stage, 2-sample Mendelian randomization study
Source: Medicine (Baltimore). 2026 Feb 6;105(6):e47598. doi: 10.1097/MD.0000000000047598 (PMC12885695; doi:10.1097/MD.0000000000047598)
Supplement: Supplementary file 3 [file medi-105-e47598-s003.docx]

**Figure. S1** Forest plots for 20 blood metabolites with significant associations with AS identified by primary analysis and sensitivity analysis.

**
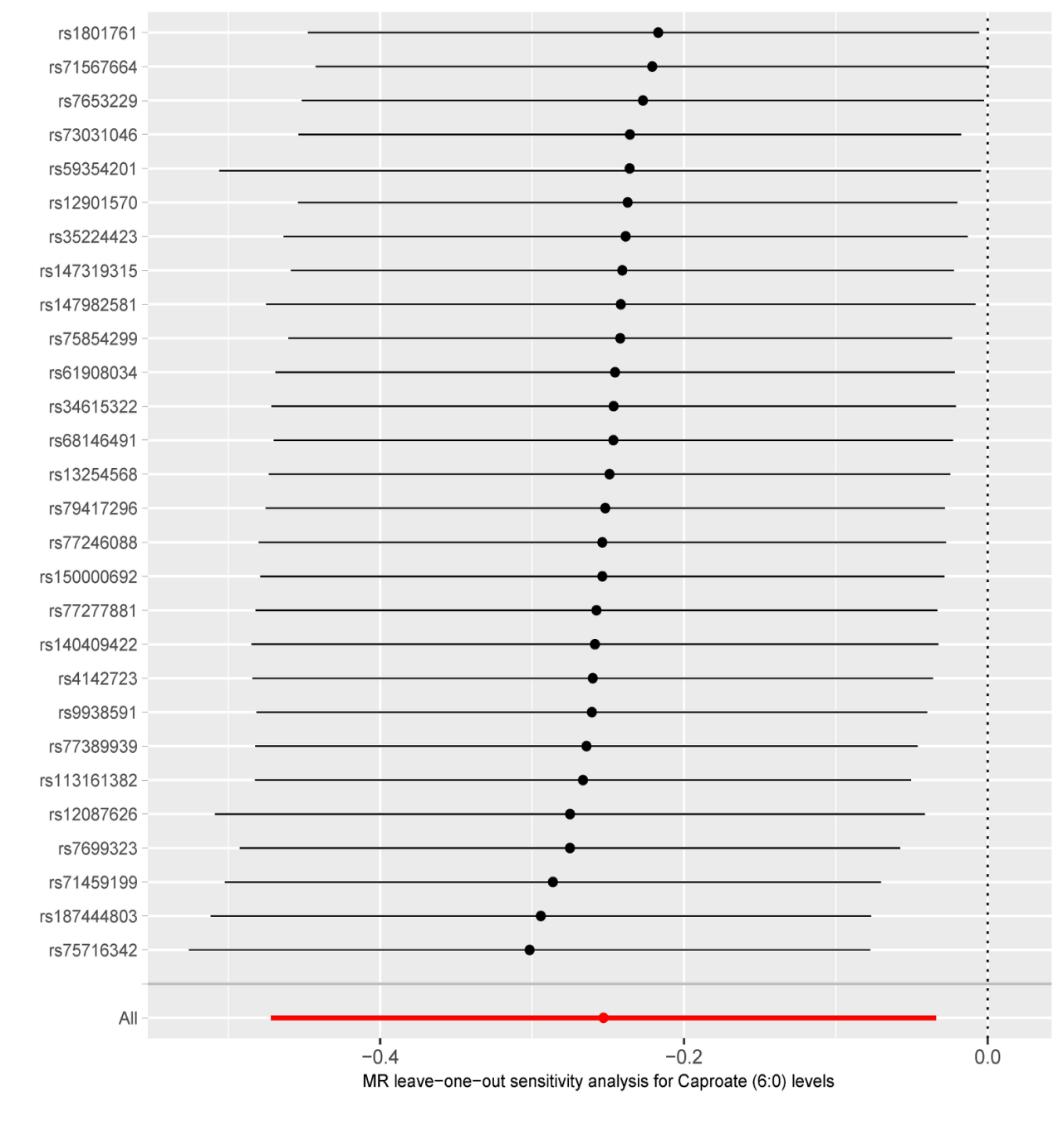
**

Funnel plot of genetic association estimates for Caproate (6:0) levels on AS

**
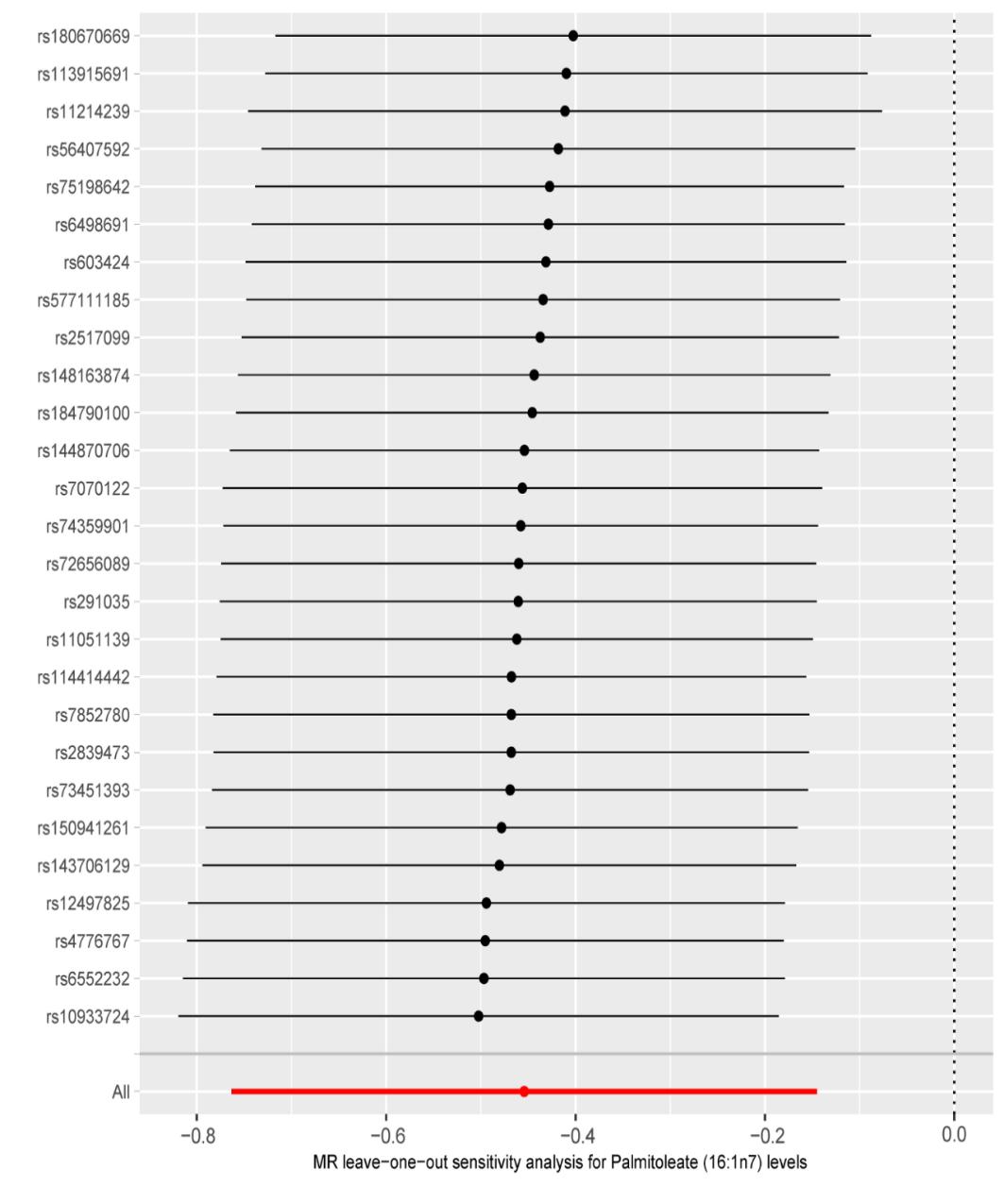
**

Funnel plot of genetic association estimates for Palmitoleate (16:1n7) levels on AS

**
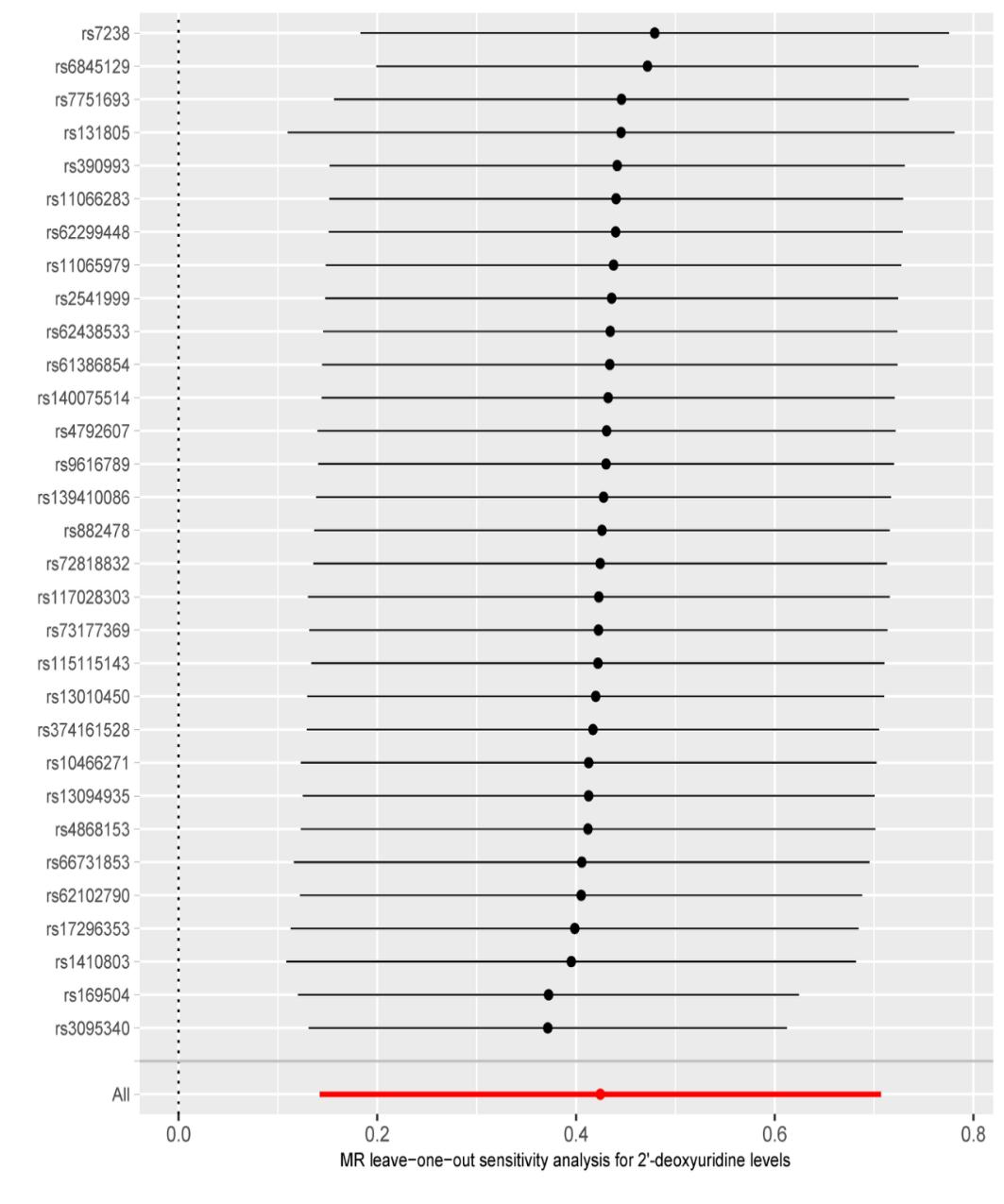
**

Funnel plot of genetic association estimates for 2'-deoxyuridine levels on AS

**
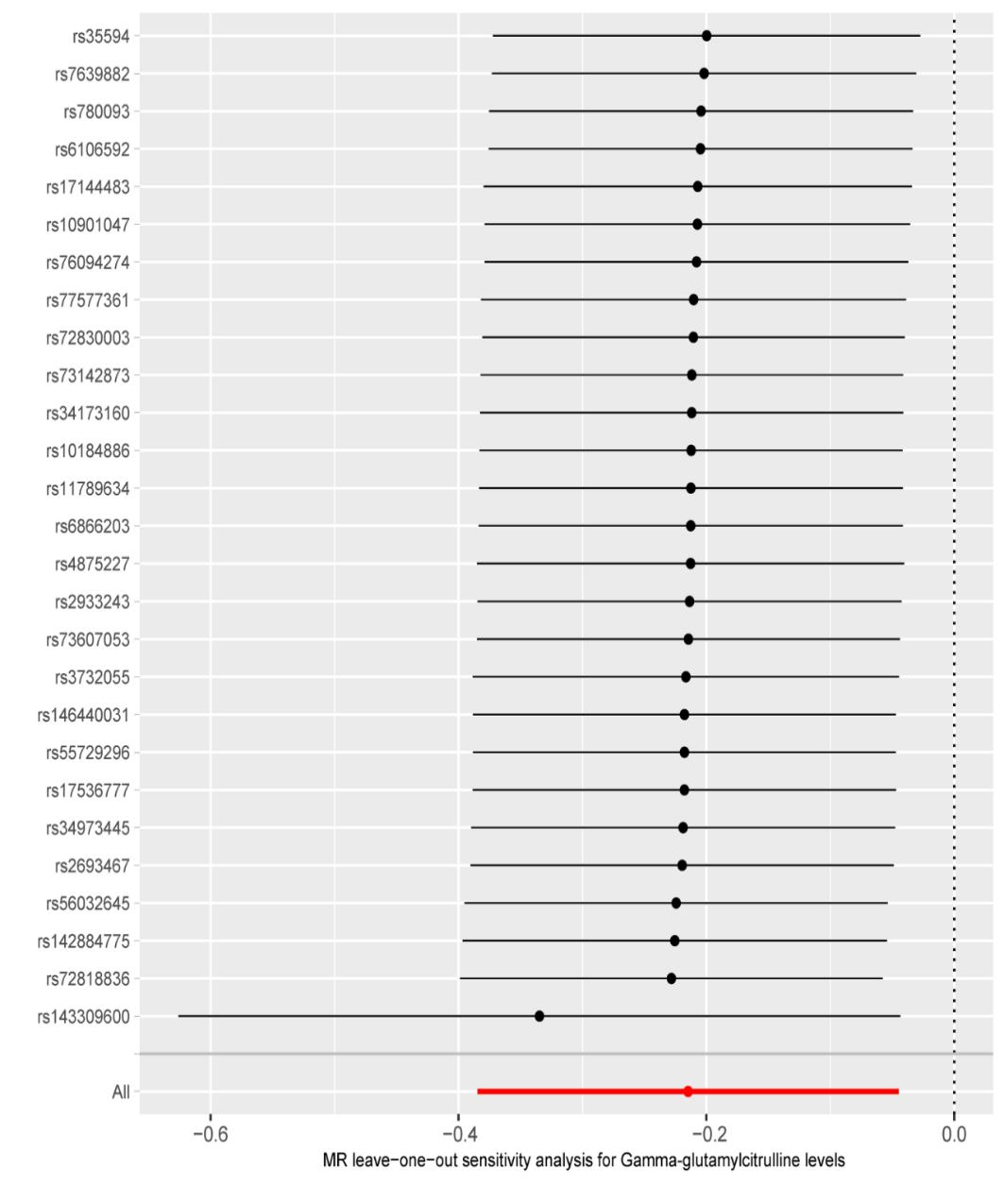
**

Funnel plot of genetic association estimates for Gamma-glutamylcitrulline levels on AS

**
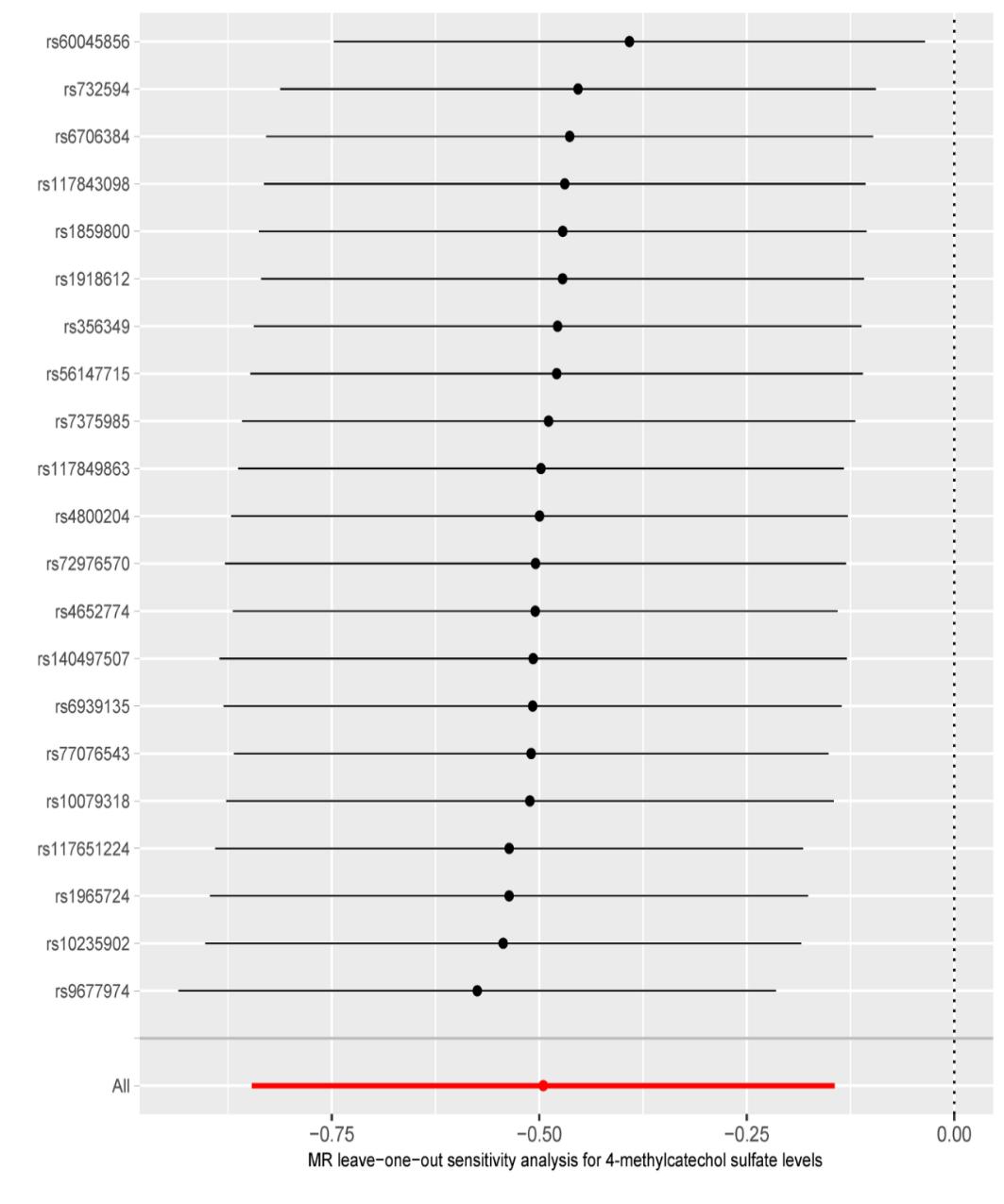
**

Funnel plot of genetic association estimates for 4-methylcatechol sulfate levels on AS

**
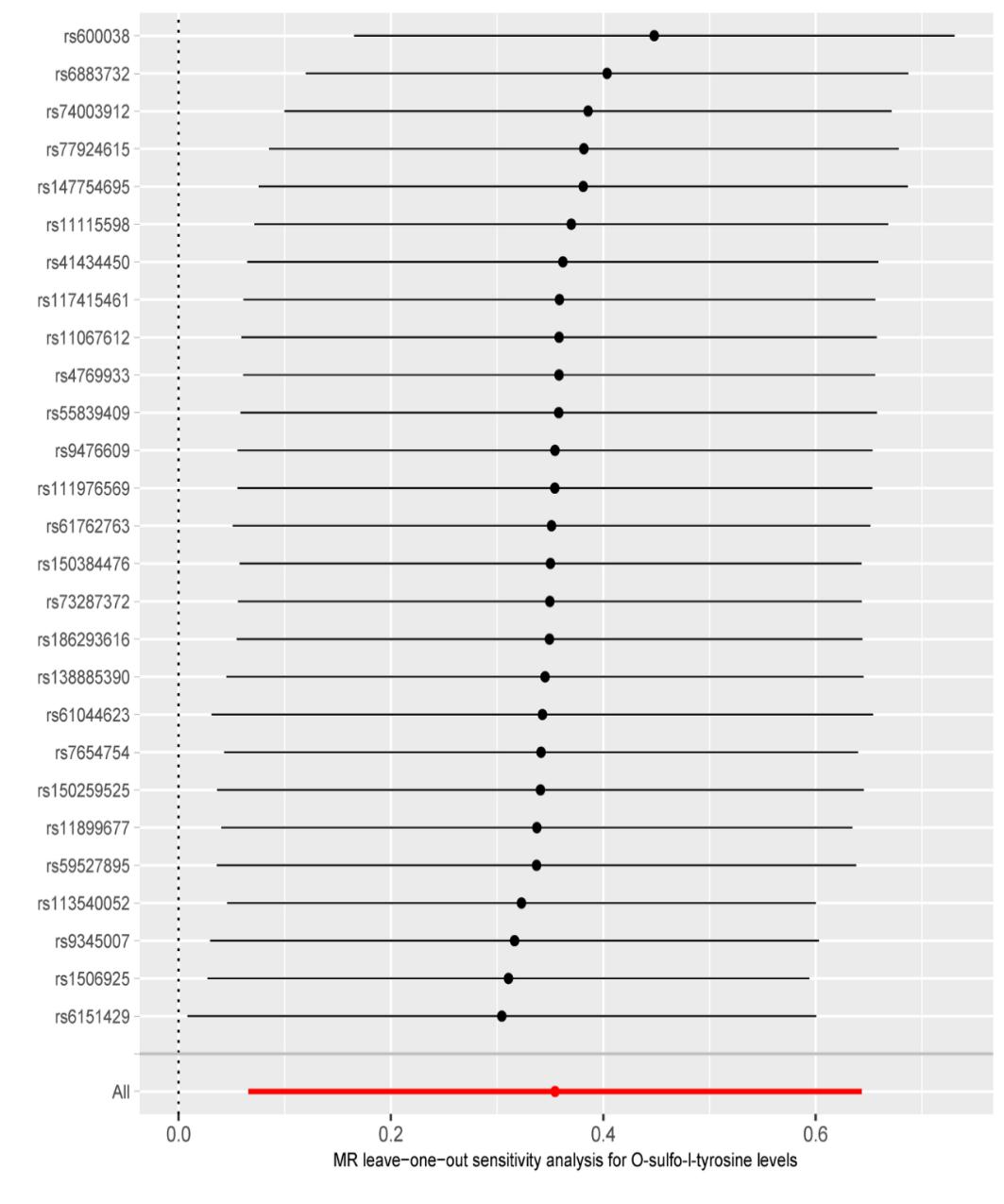
**

Funnel plot of genetic association estimates for O-sulfo-l-tyrosine levels on AS

**
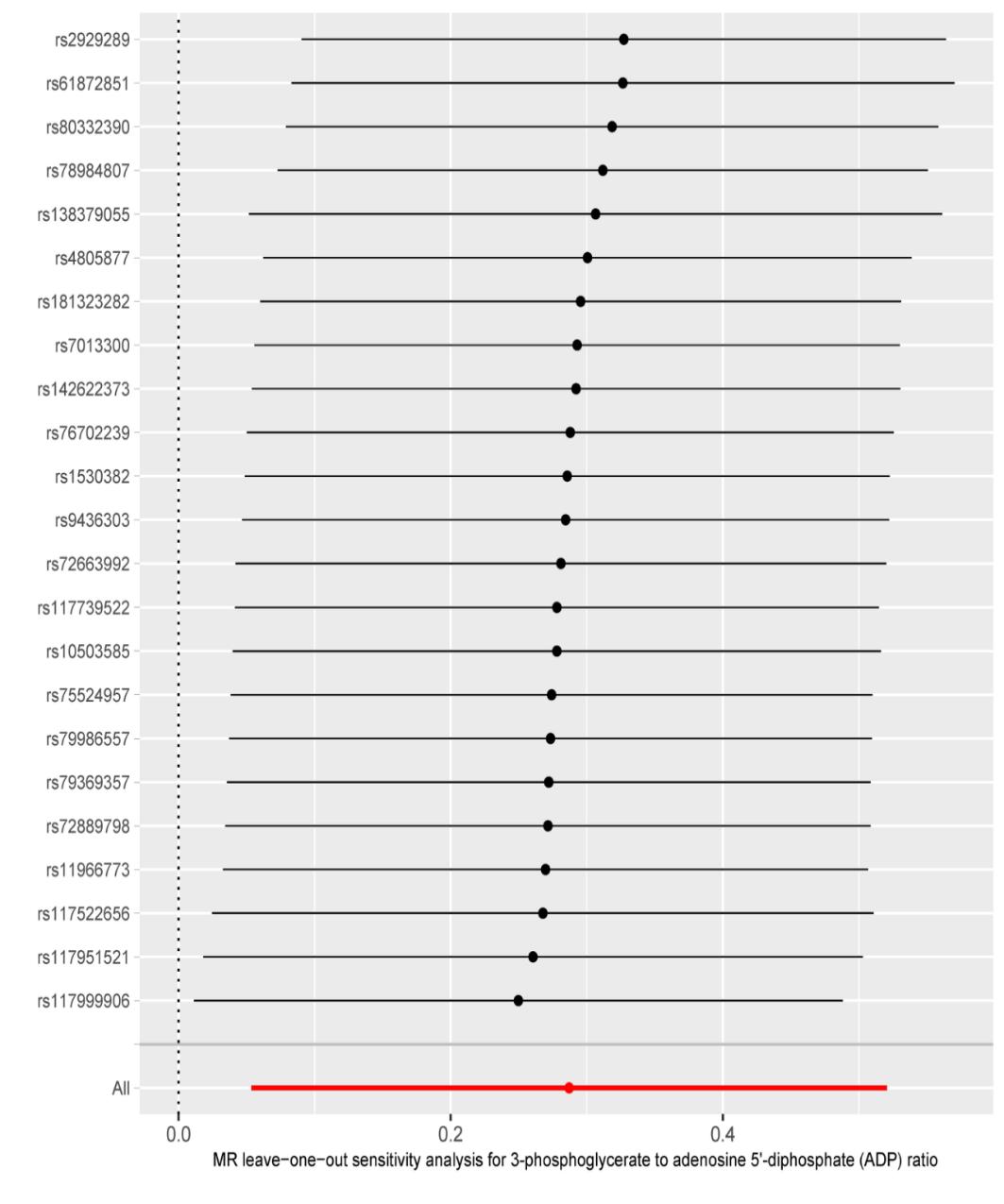
**

Funnel plot of genetic association estimates for 3-phosphoglycerate to adenosine 5'-diphosphate (ADP) ratio on AS

**
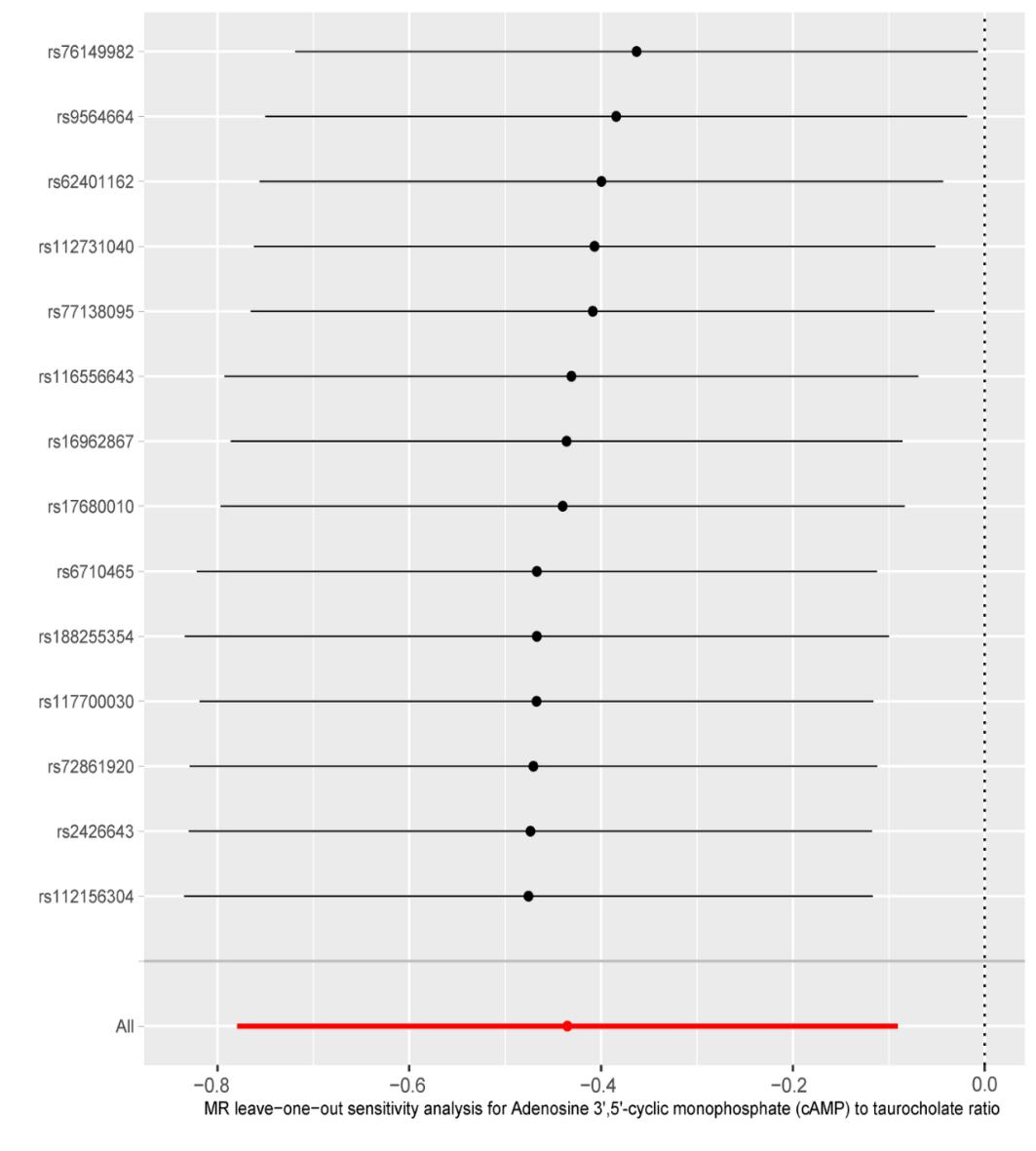
**

Funnel plot of genetic association estimates for Adenosine 3',5'-cyclic monophosphate (cAMP) to taurocholate ratio on AS

**
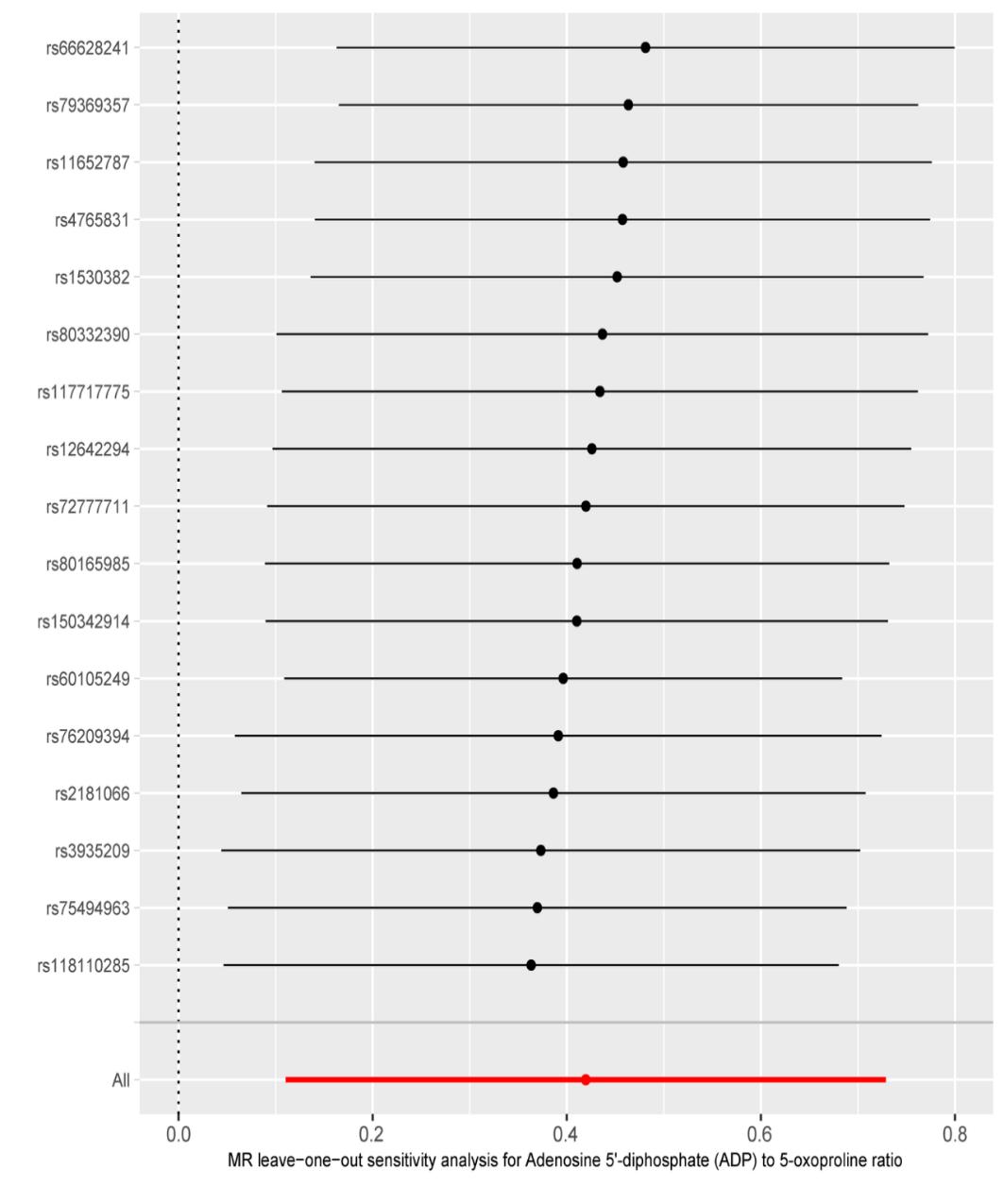
**

Funnel plot of genetic association estimates for Adenosine 5'-diphosphate (ADP) to 5-oxoproline ratio on AS

**
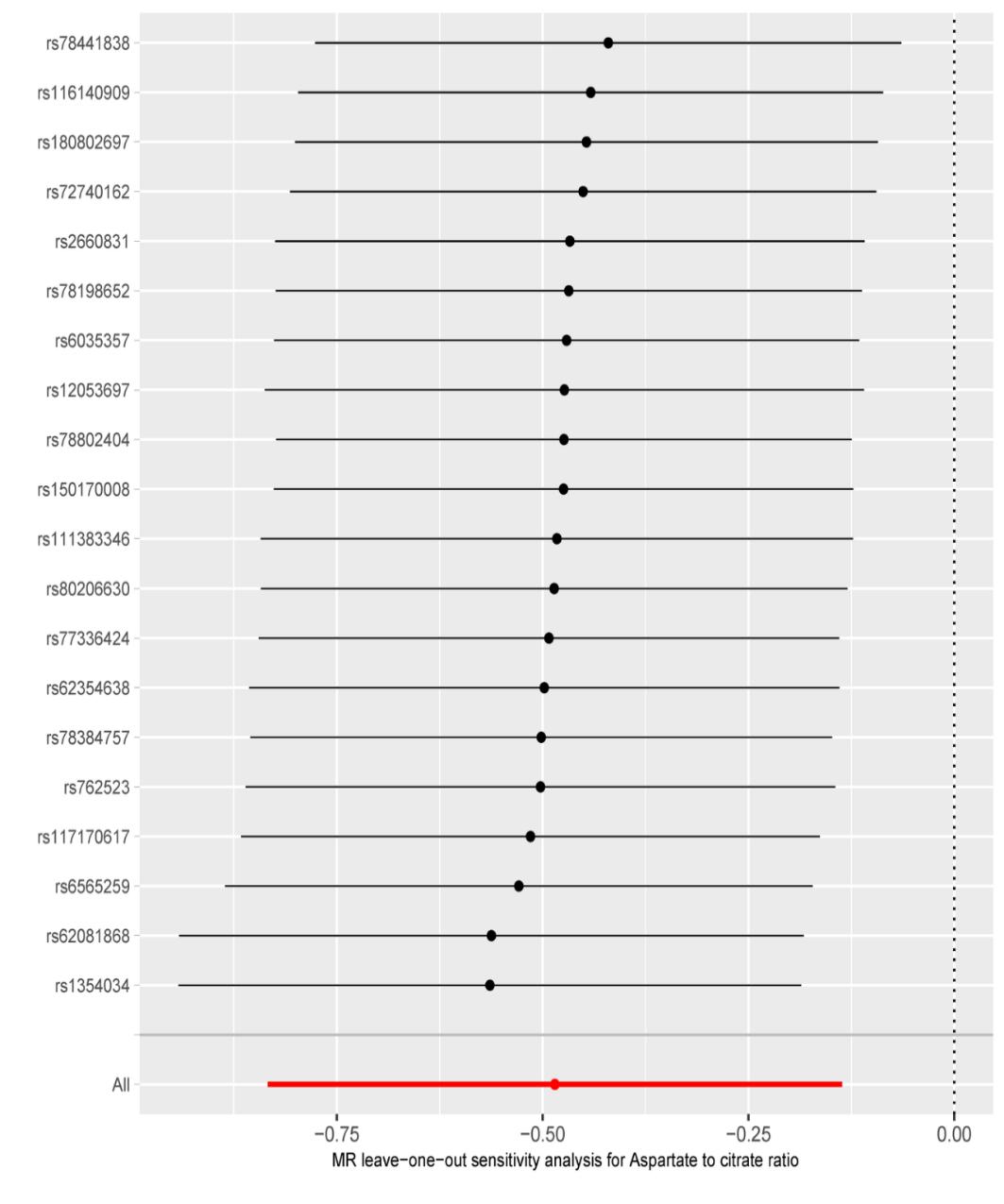
**

Funnel plot of genetic association estimates for Aspartate to citrate ratio on AS

**
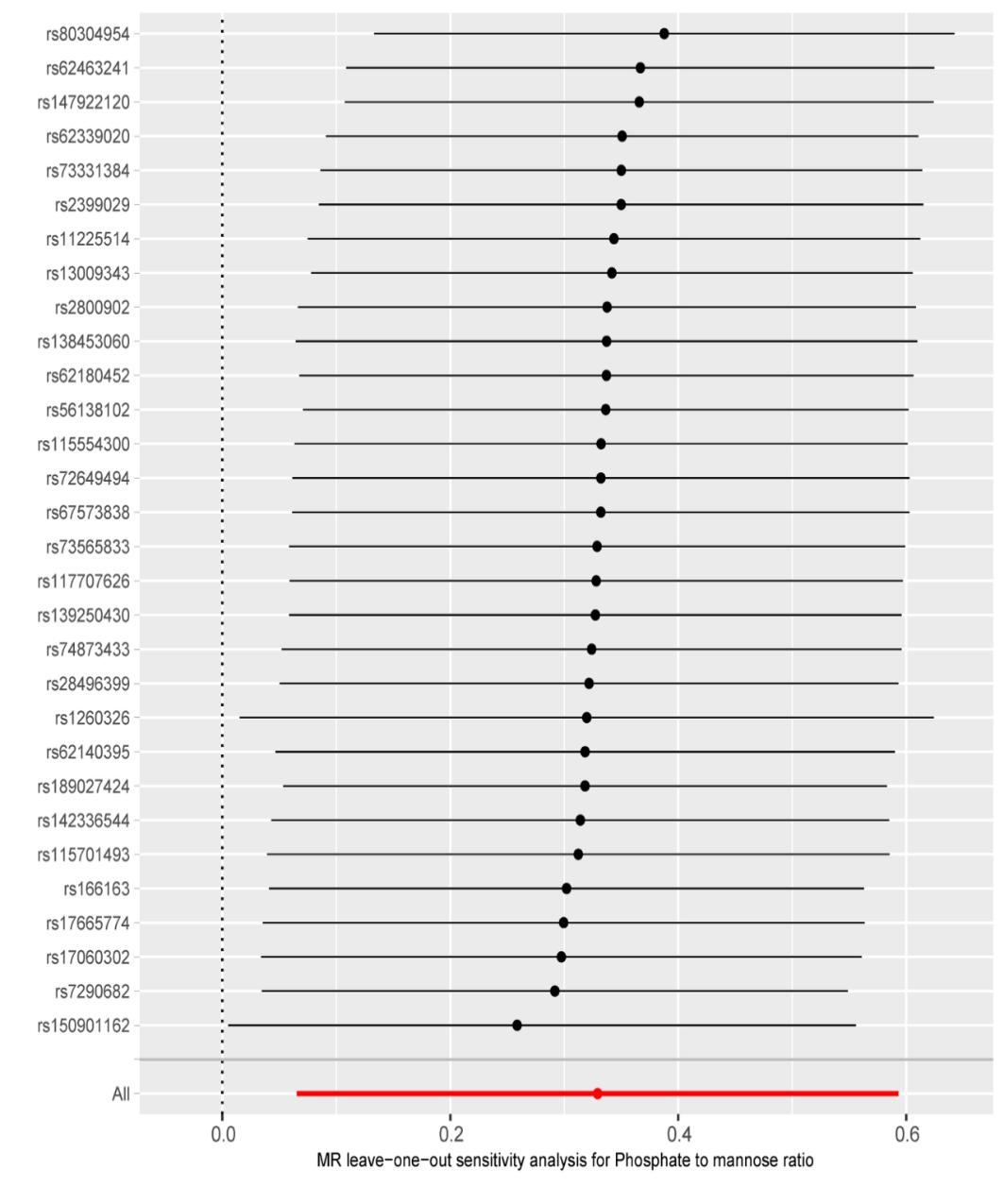
**

Funnel plot of genetic association estimates for Phosphate to mannose ratio on AS

**
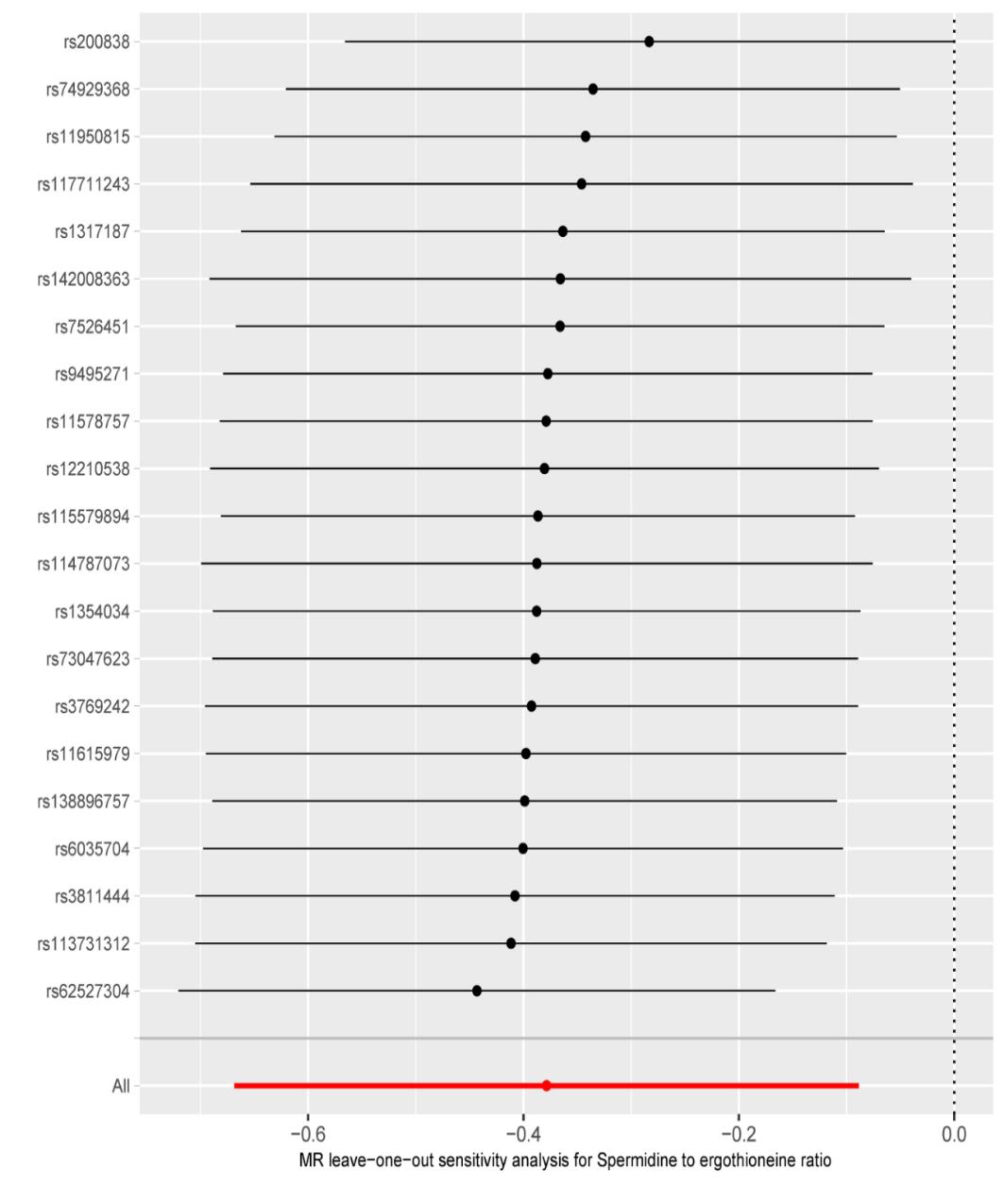
**

Funnel plot of genetic association estimates for Spermidine to ergothioneine ratio on AS

**
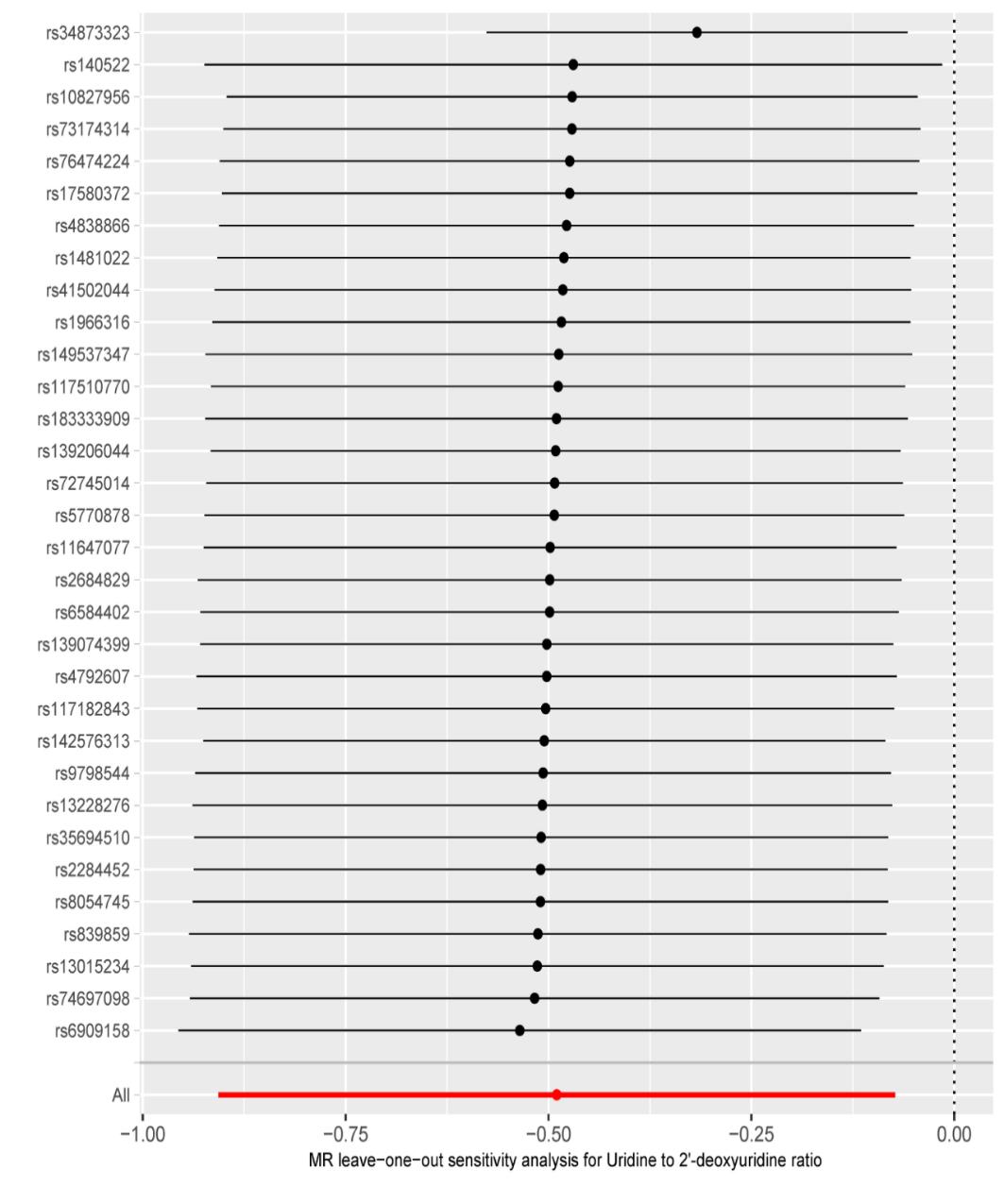
**

Funnel plot of genetic association estimates for Uridine to 2'-deoxyuridine ratio on AS

**
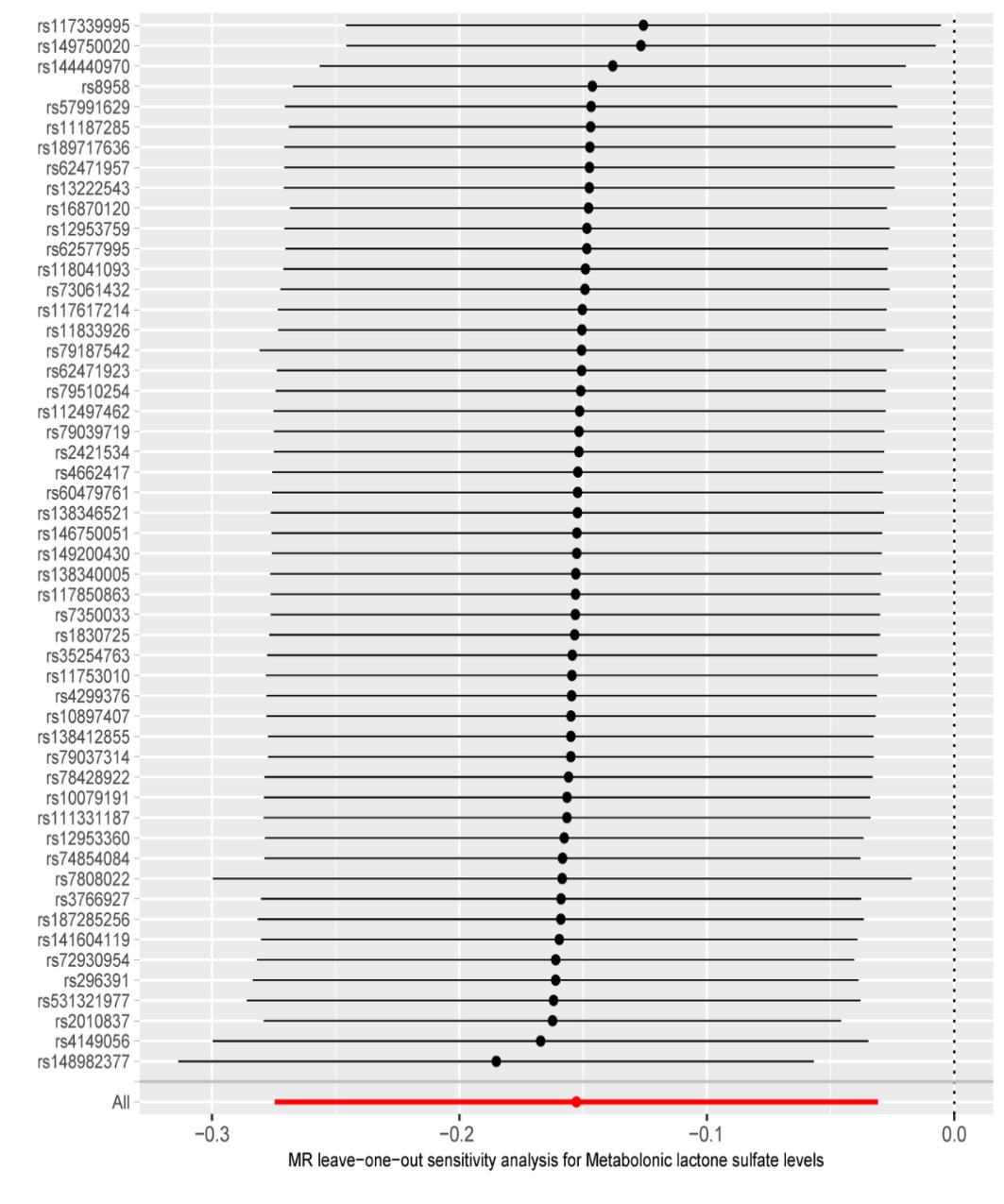
**

Funnel plot of genetic association estimates for Metabolonic lactone sulfate levels on AS

**
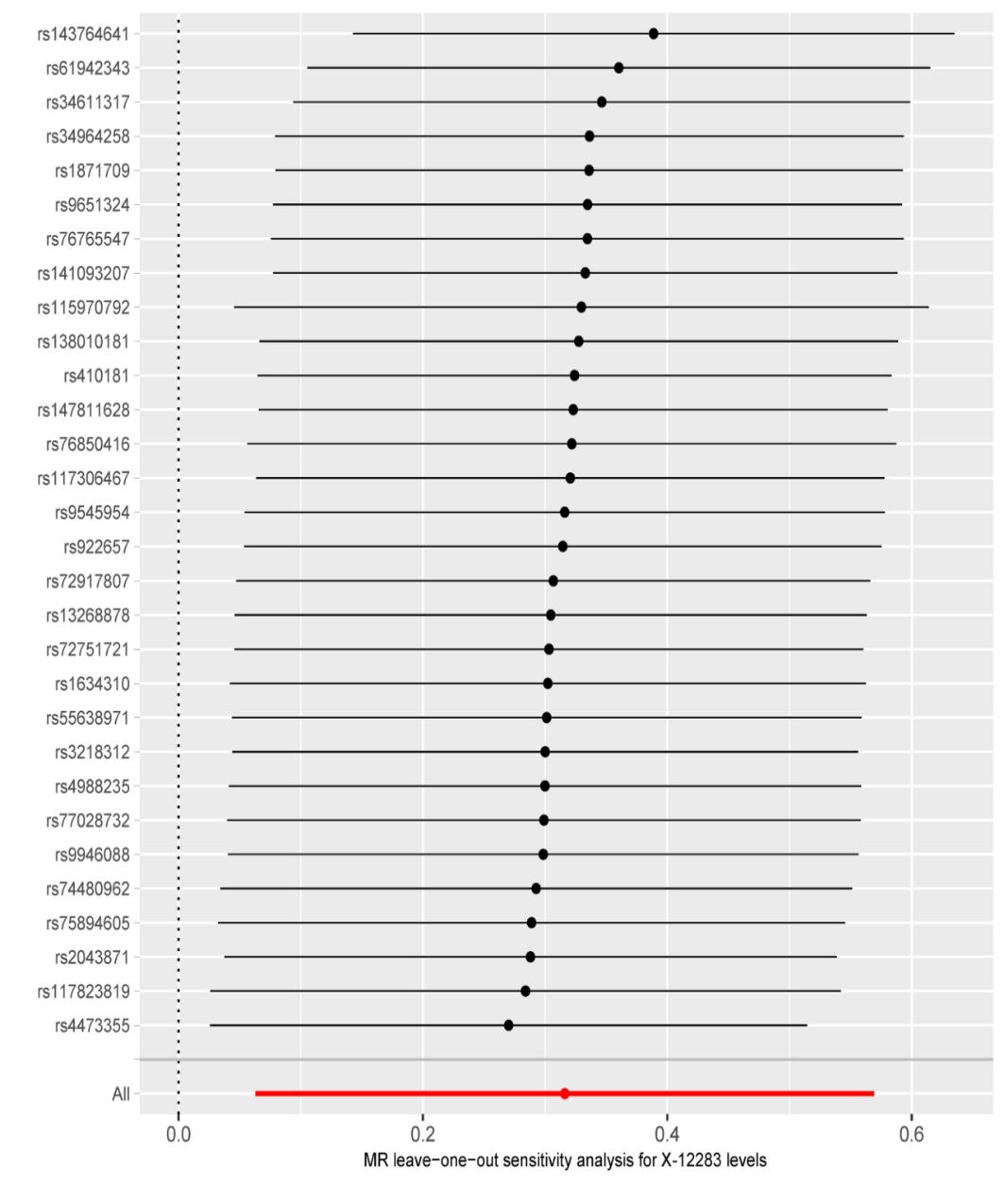
**

Funnel plot of genetic association estimates for X-12283 levels on AS

**
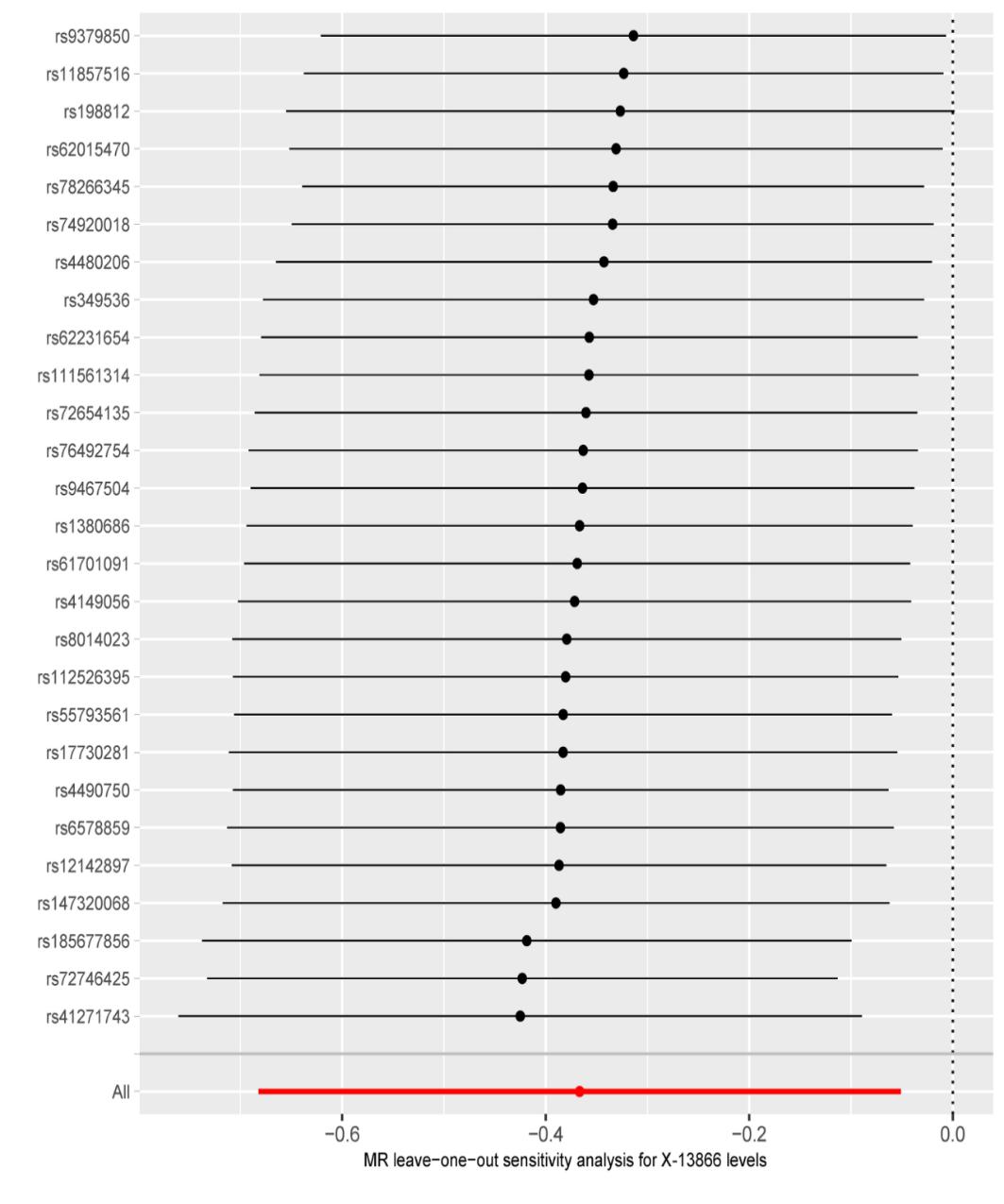
**

Funnel plot of genetic association estimates for X-13866 levels on AS

**
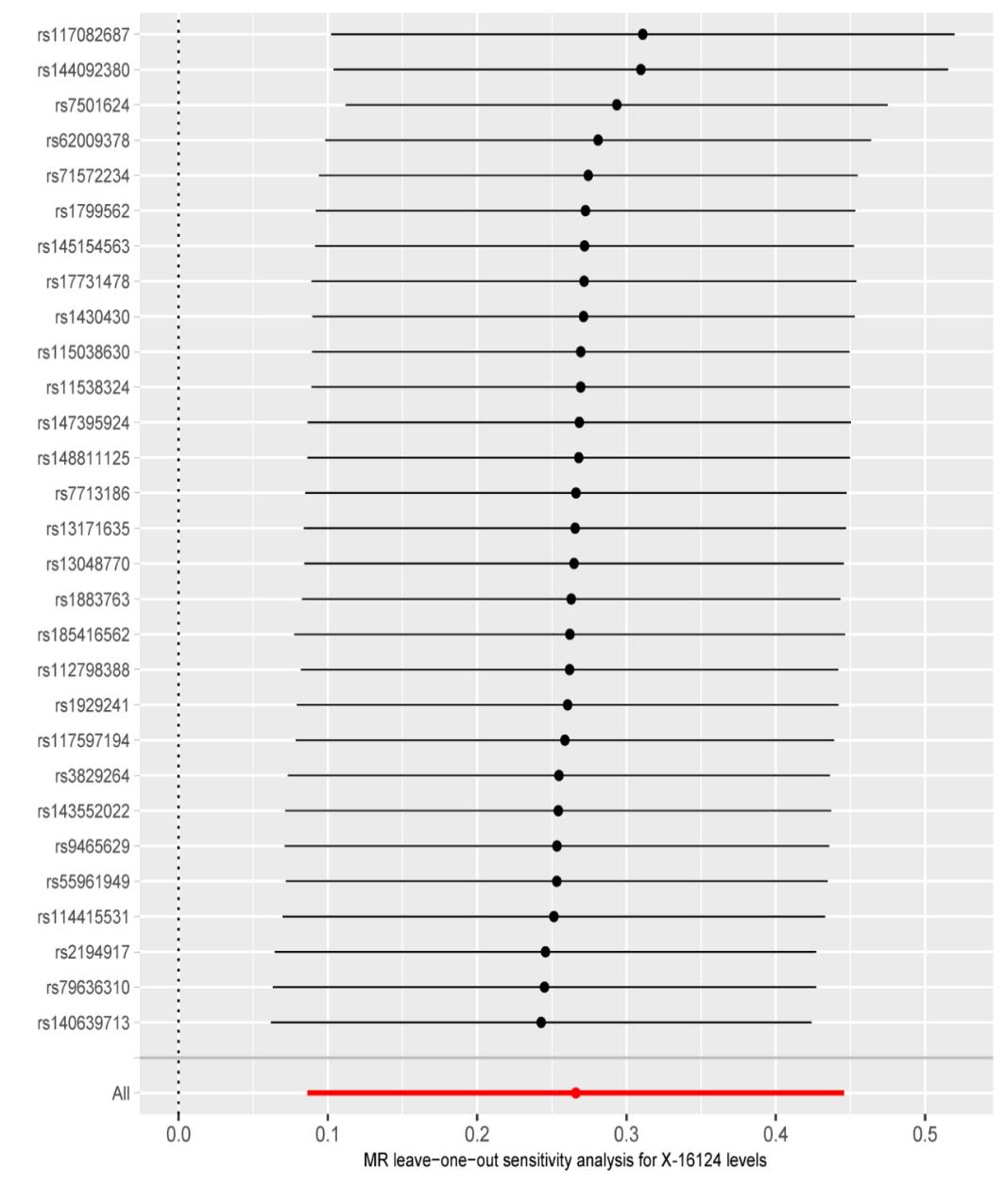
**

Funnel plot of genetic association estimates for X-16124 levels on AS

**
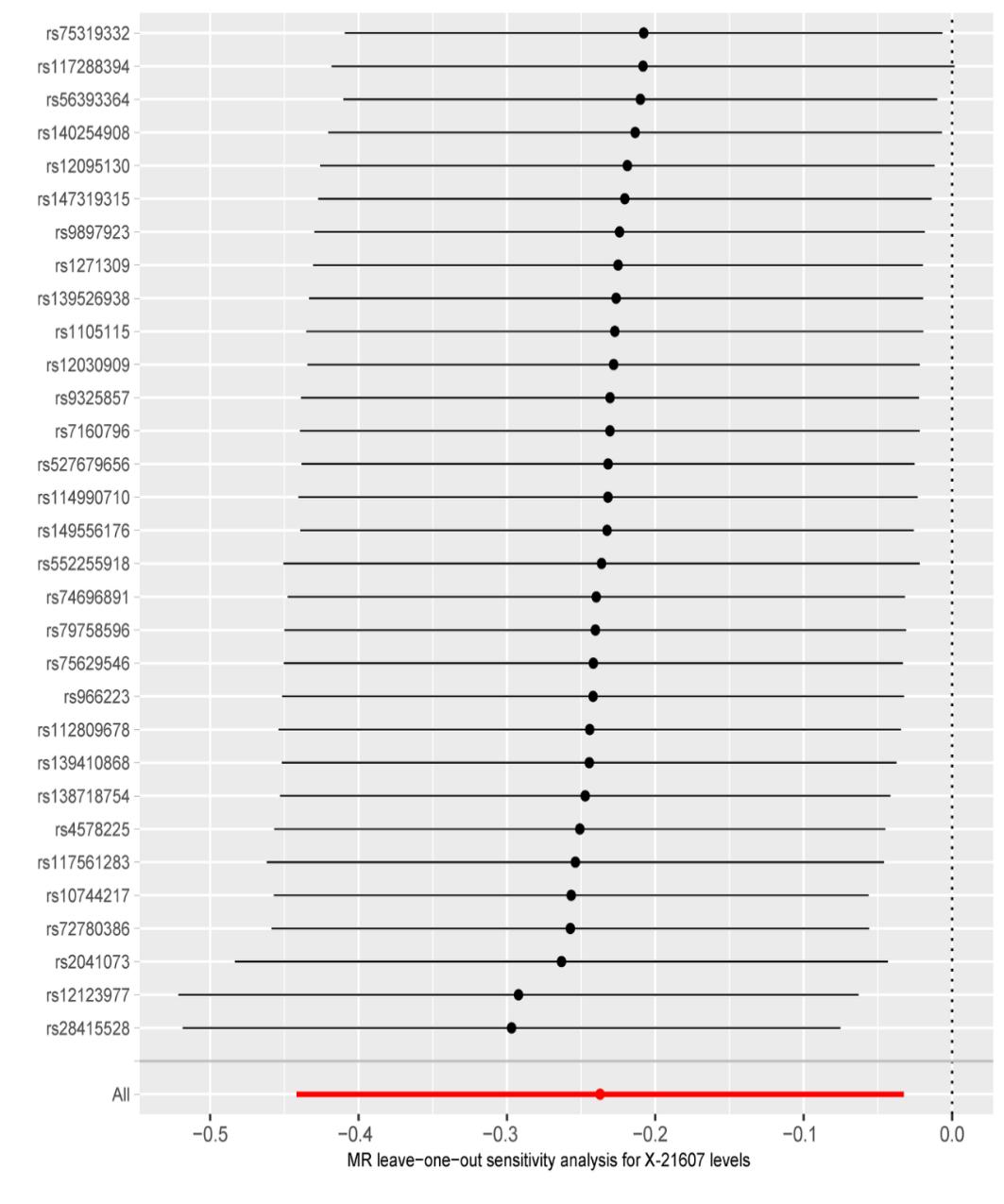
**

Funnel plot of genetic association estimates for X-21607 levels on AS

**
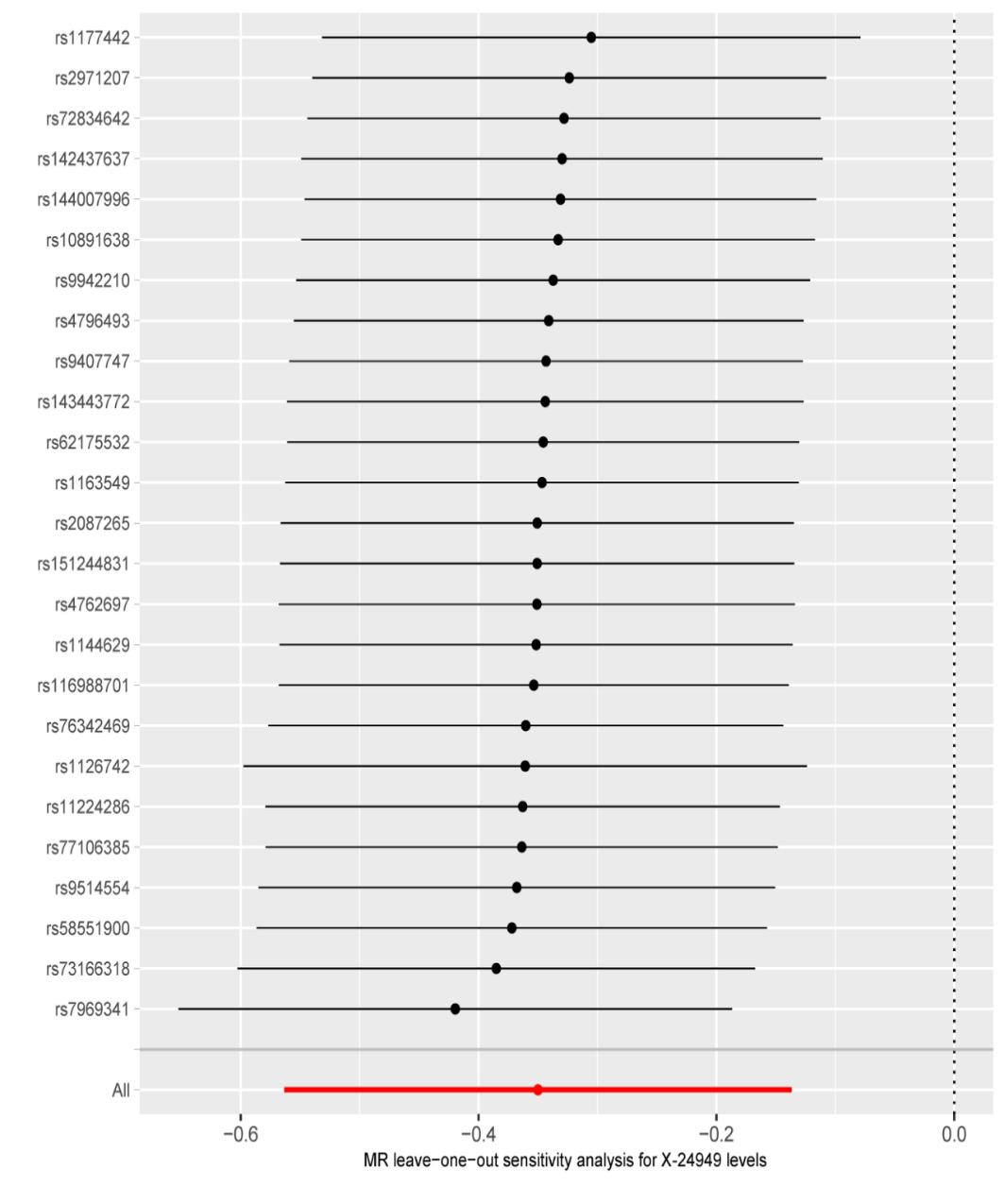
**

Funnel plot of genetic association estimates for X-24949 levels on AS

**
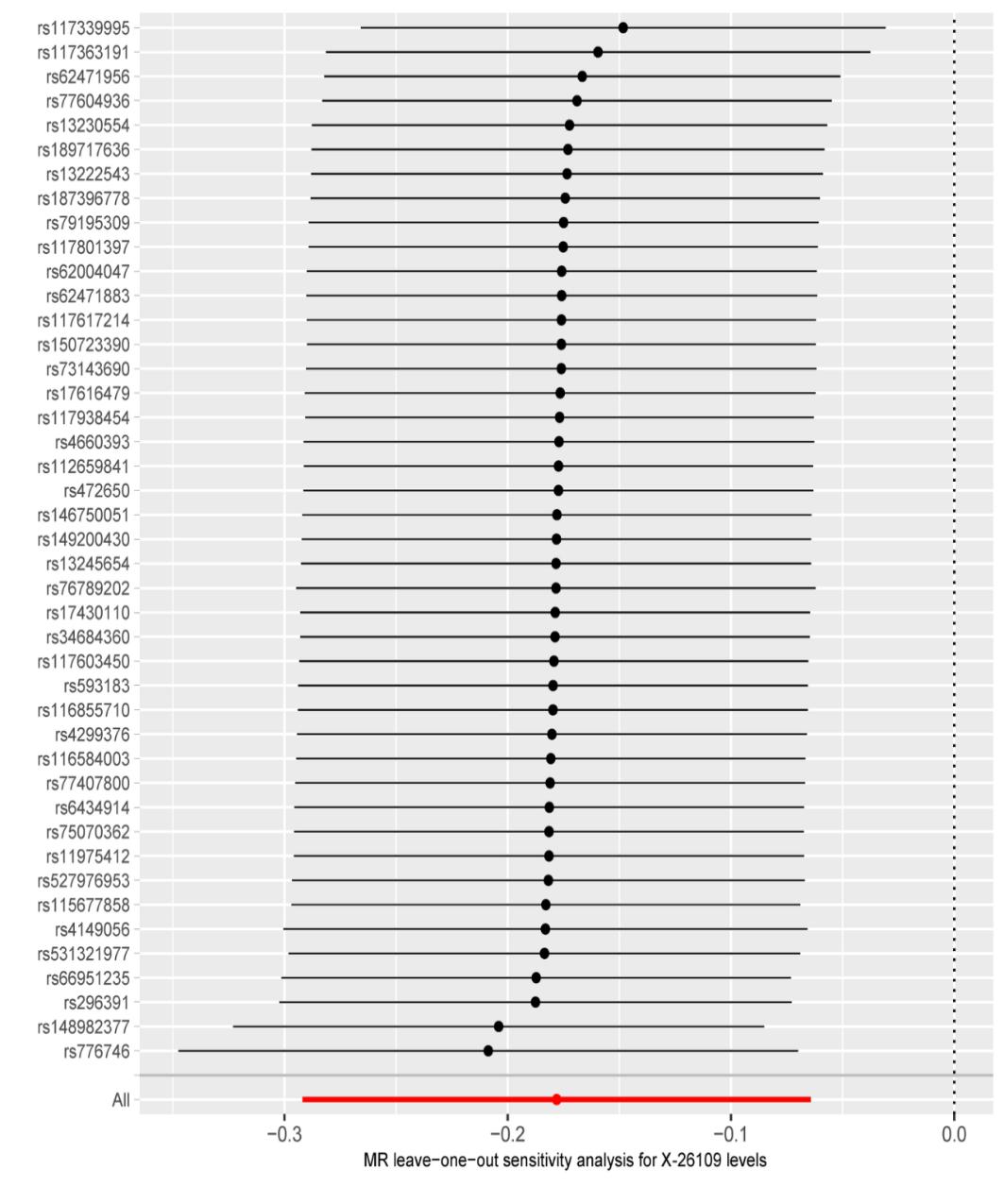
**

Funnel plot of genetic association estimates for X-26109 levels on AS

**Figure. S2** Funnel plots for 20 blood metabolites with significant associations with AS identified by primary analysis and sensitivity analysis.


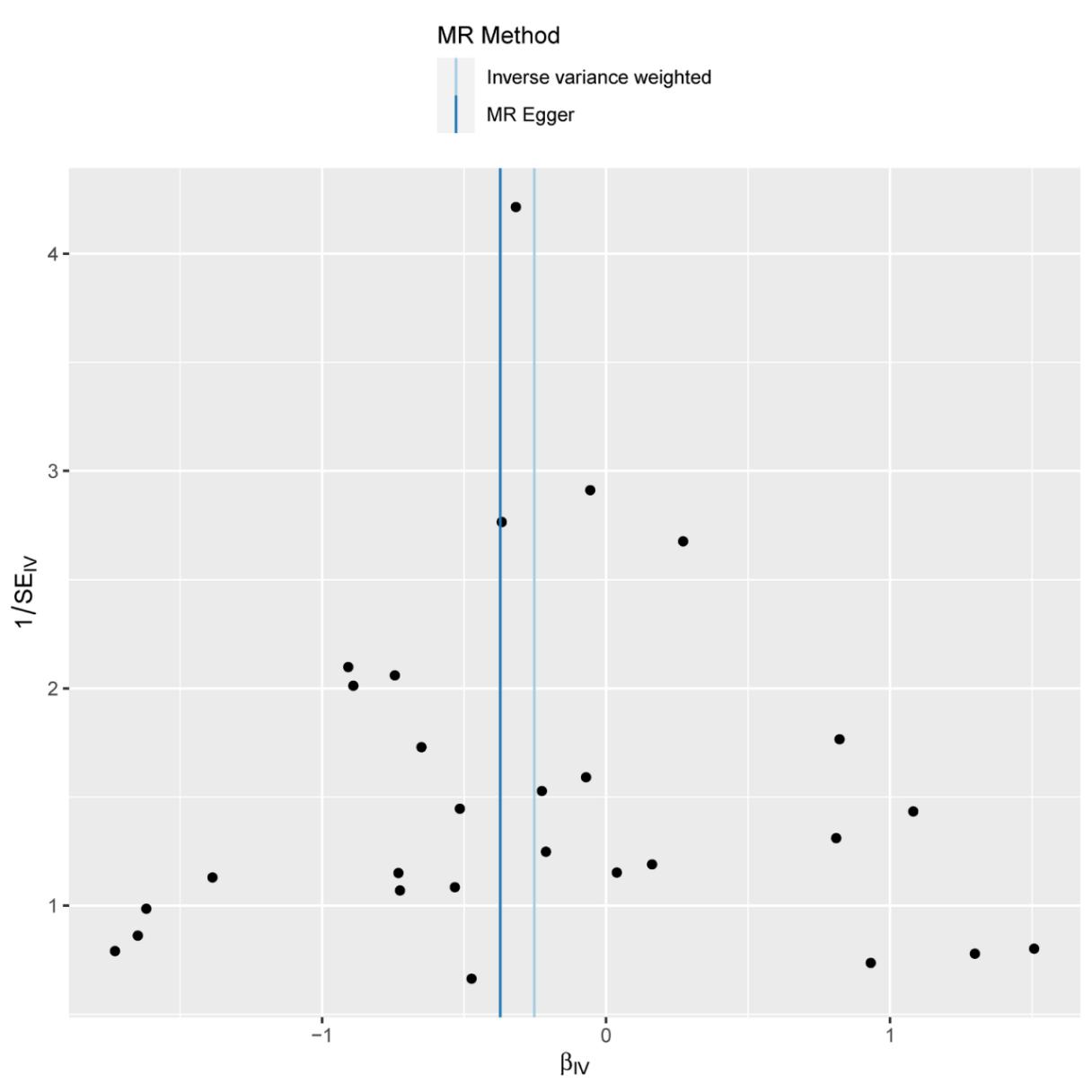


Funnel plot of genetic association estimates for Caproate (6:0) levels


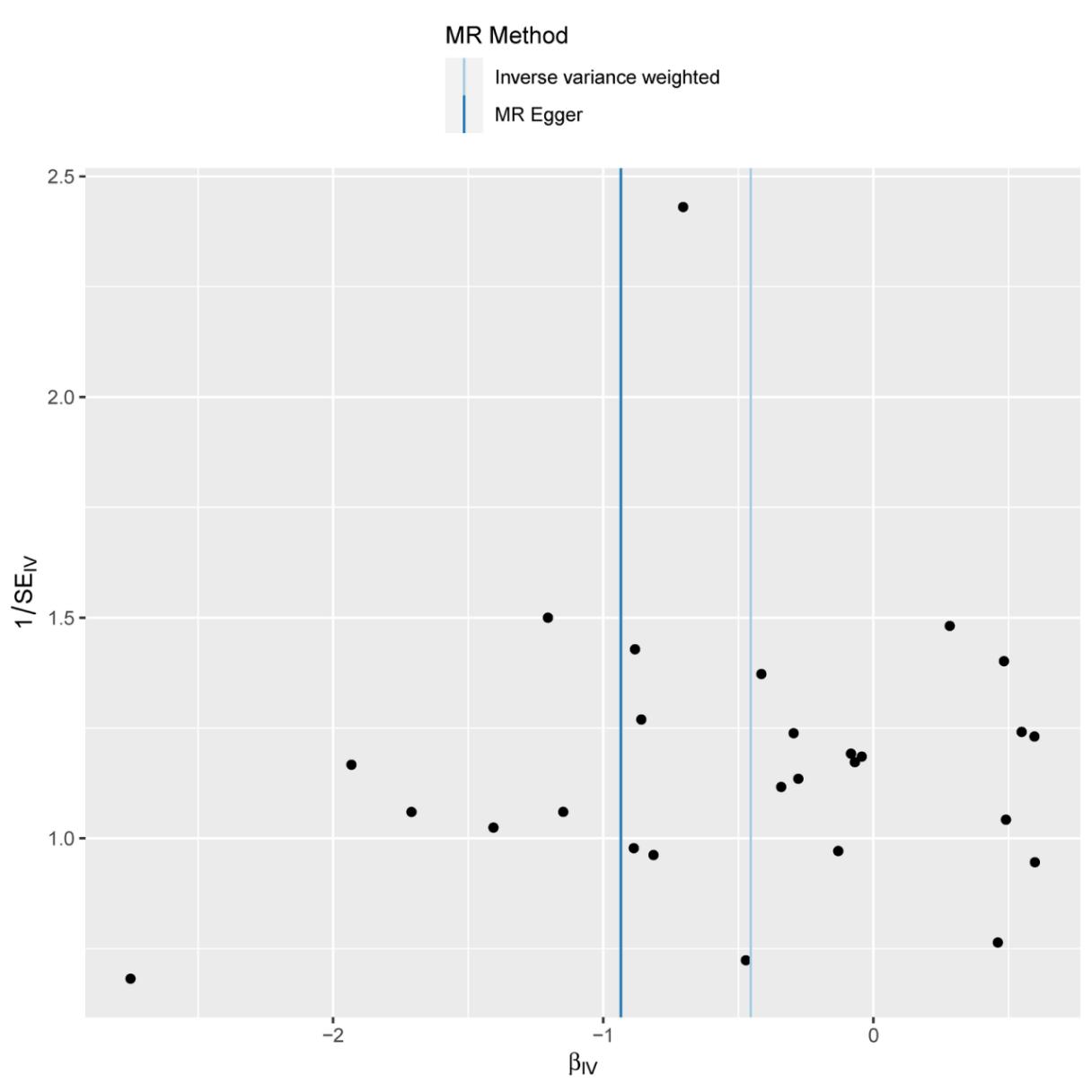


Funnel plot of genetic association estimates for Palmitoleate (16:1n7) levels


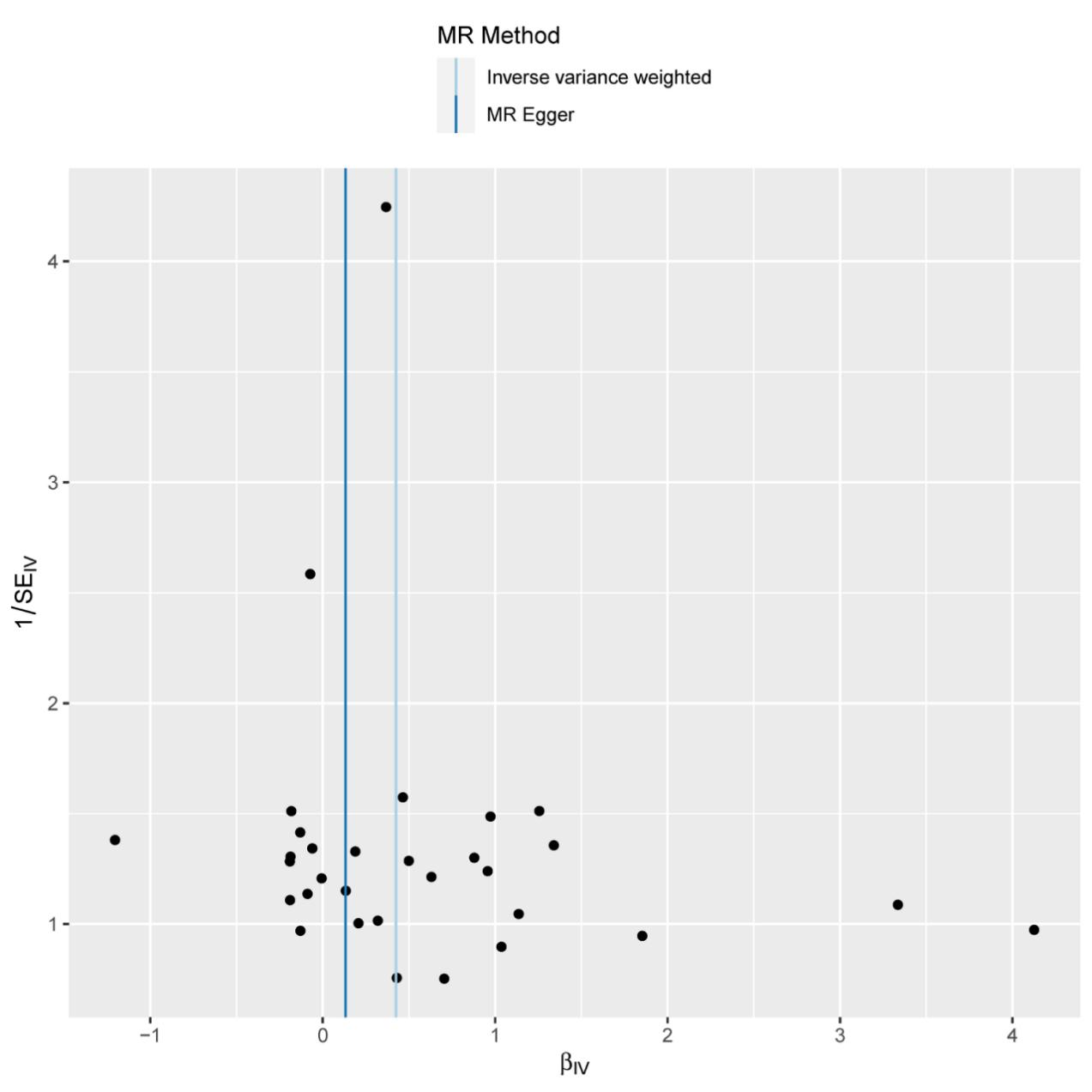


Funnel plot of genetic association estimates for 2'-deoxyuridine levels
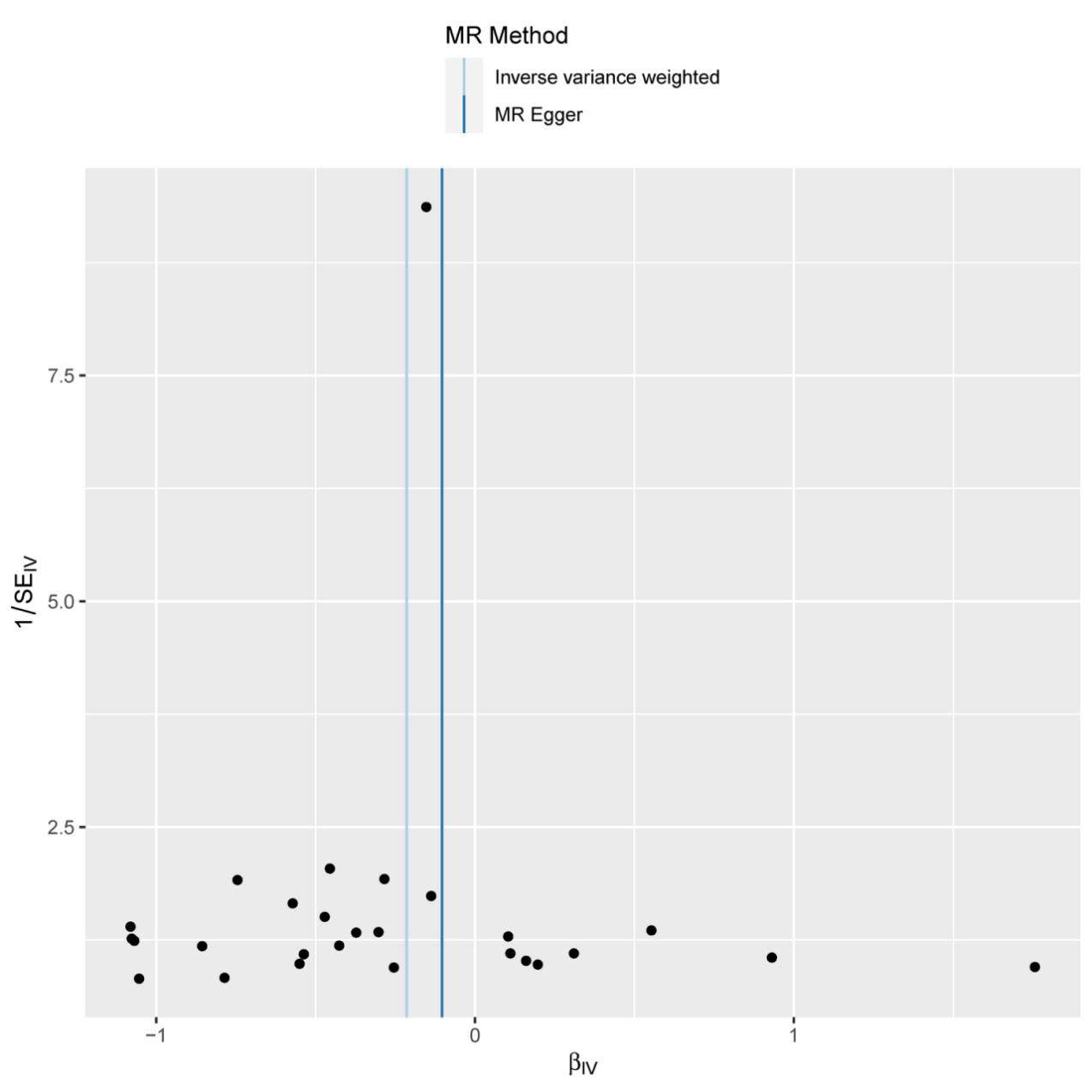


Funnel plot of genetic association estimates for Gamma-glutamylcitrulline levels
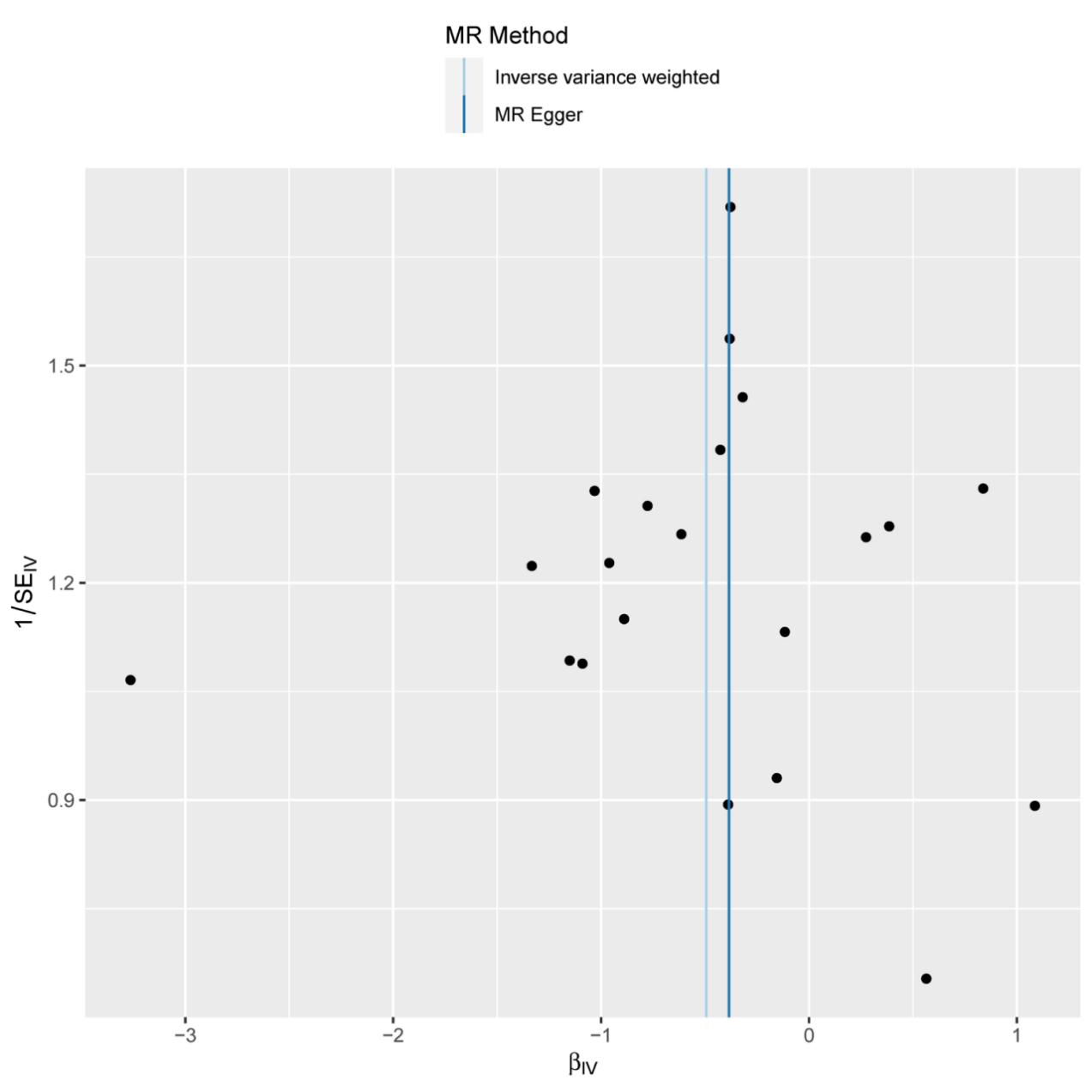


Funnel plot of genetic association estimates for 4-methylcatechol sulfate levels
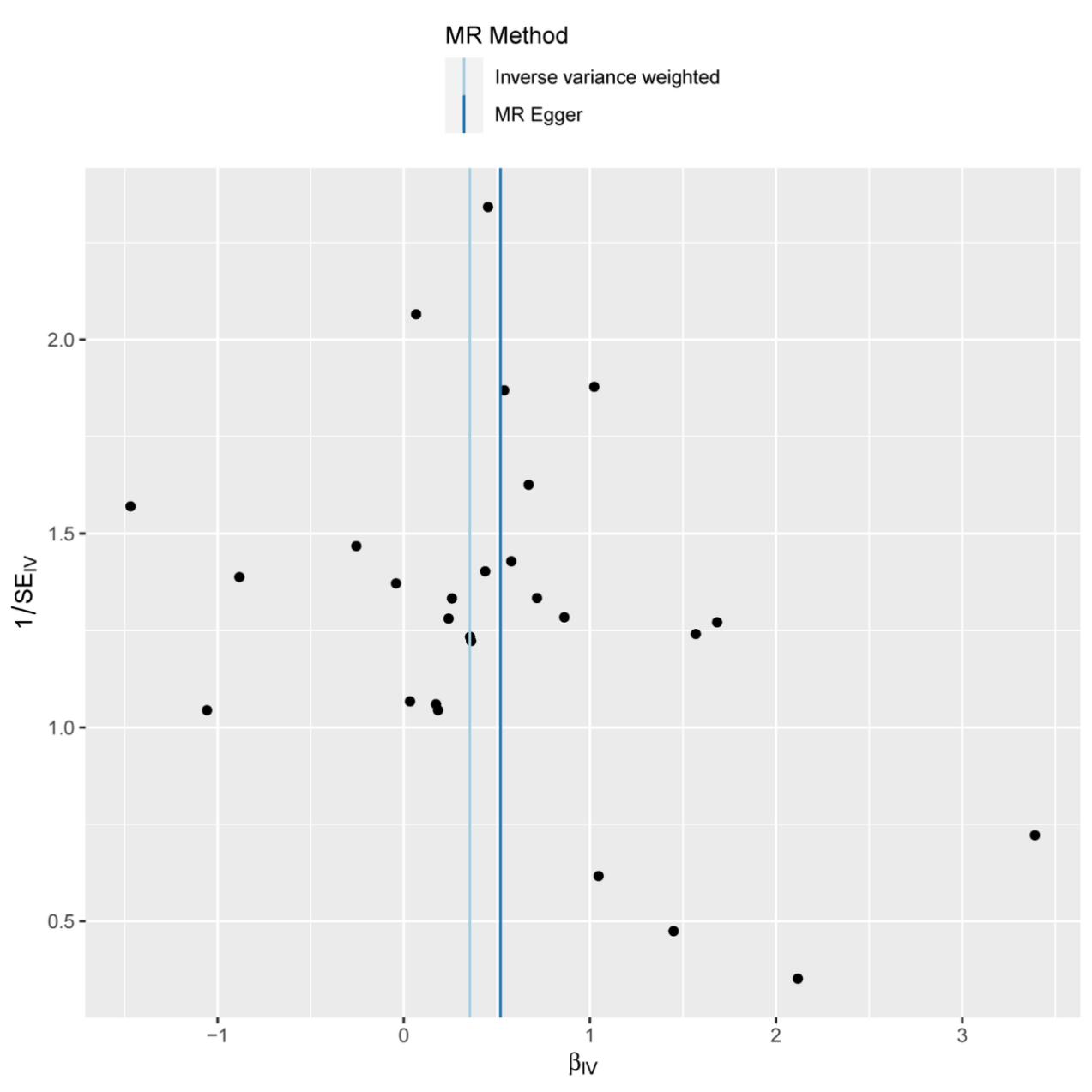


Funnel plot of genetic association estimates for O-sulfo-l-tyrosine levels
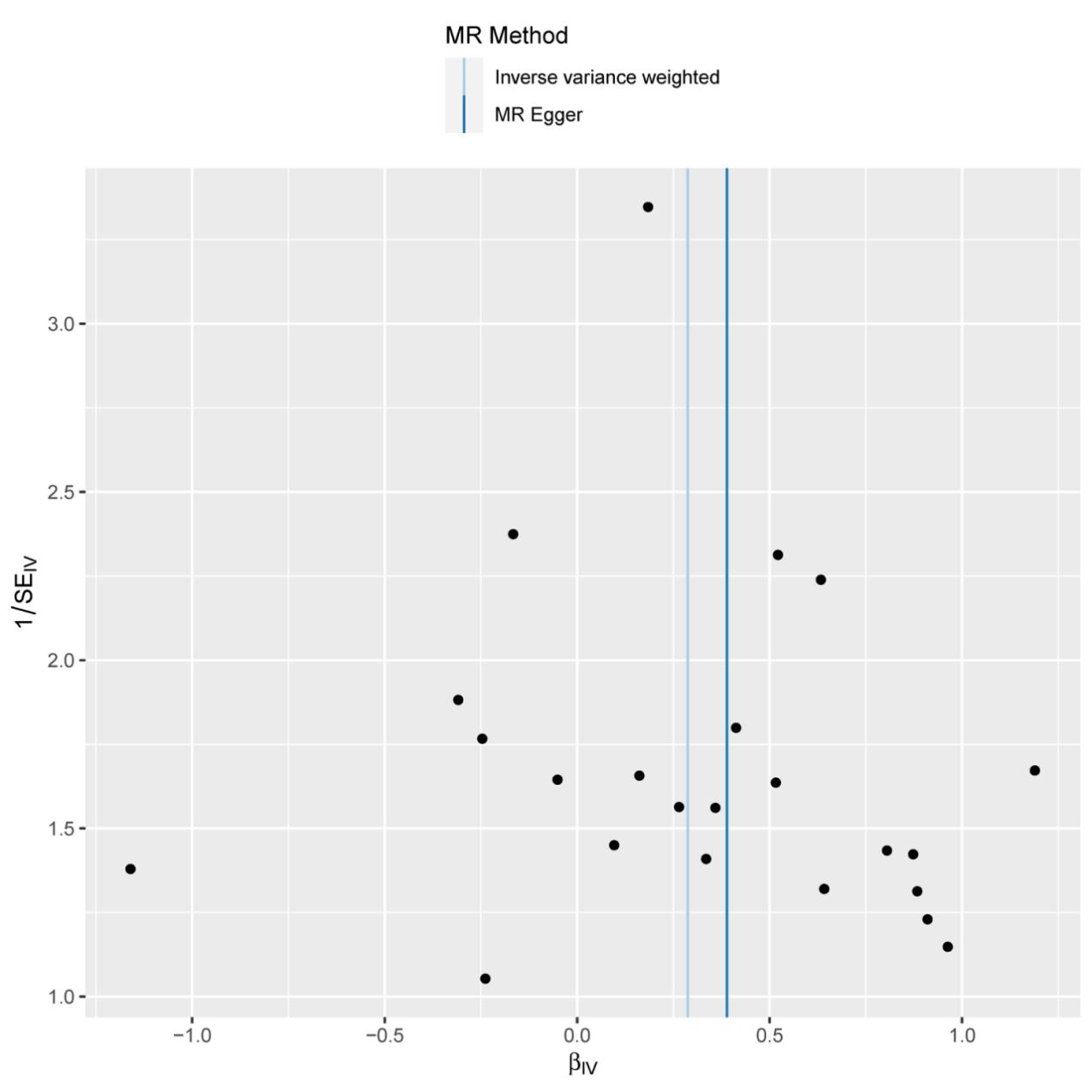


Funnel plot of genetic association estimates for 3-phosphoglycerate to adenosine 5'-diphosphate (ADP) ratio
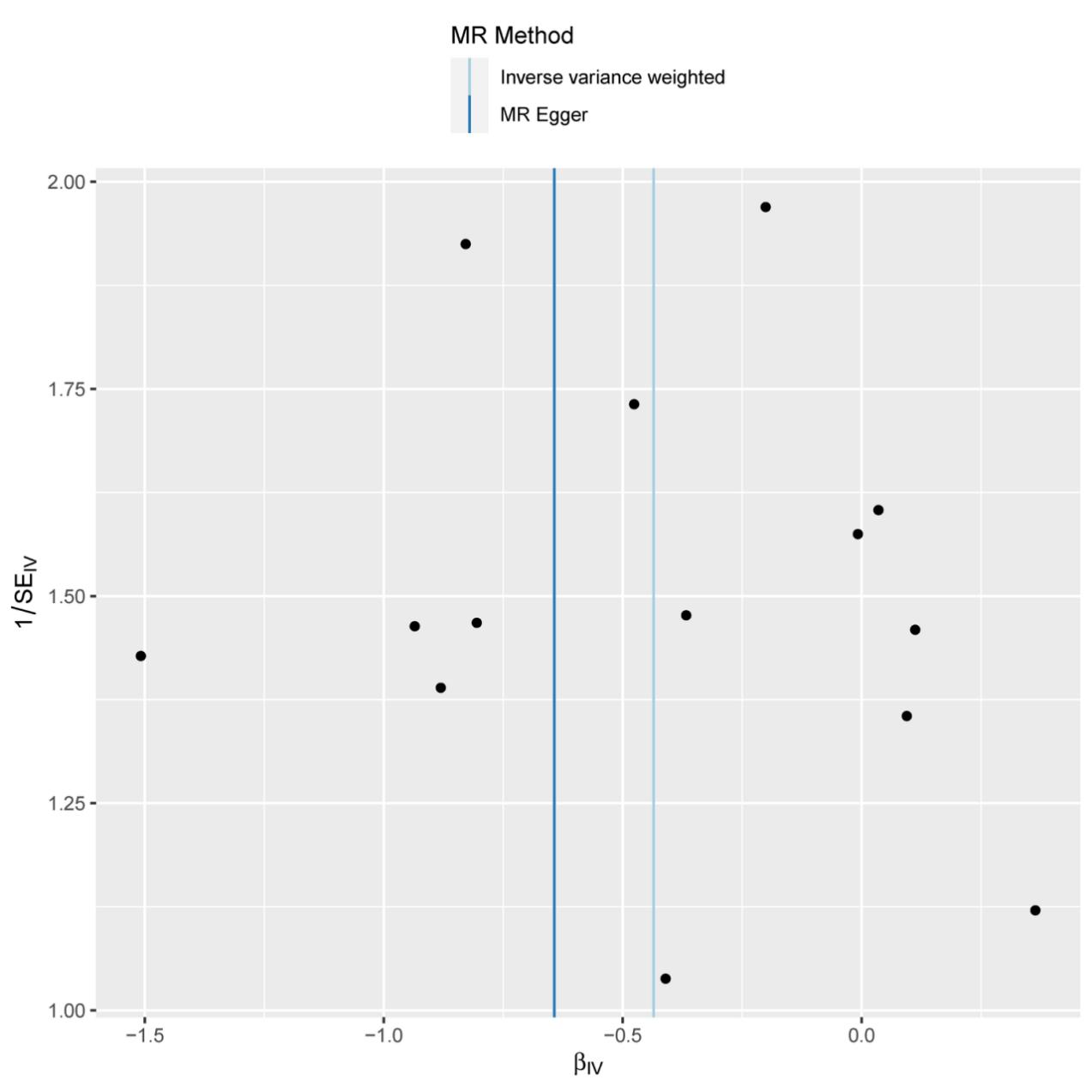


Funnel plot of genetic association estimates for Adenosine 3',5'-cyclic monophosphate (cAMP) to taurocholate ratio
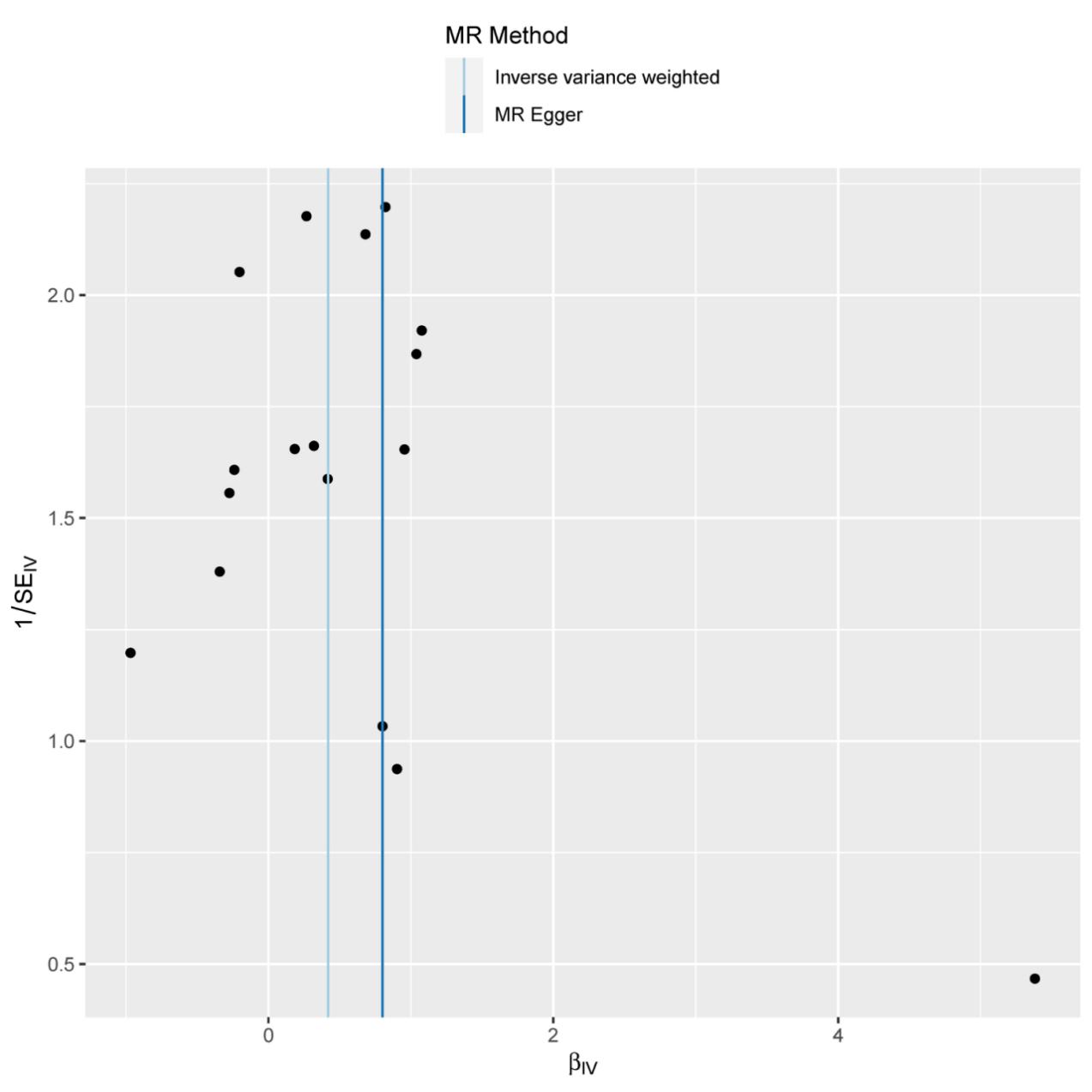


Funnel plot of genetic association estimates for Adenosine 5'-diphosphate (ADP) to 5-oxoproline ratio
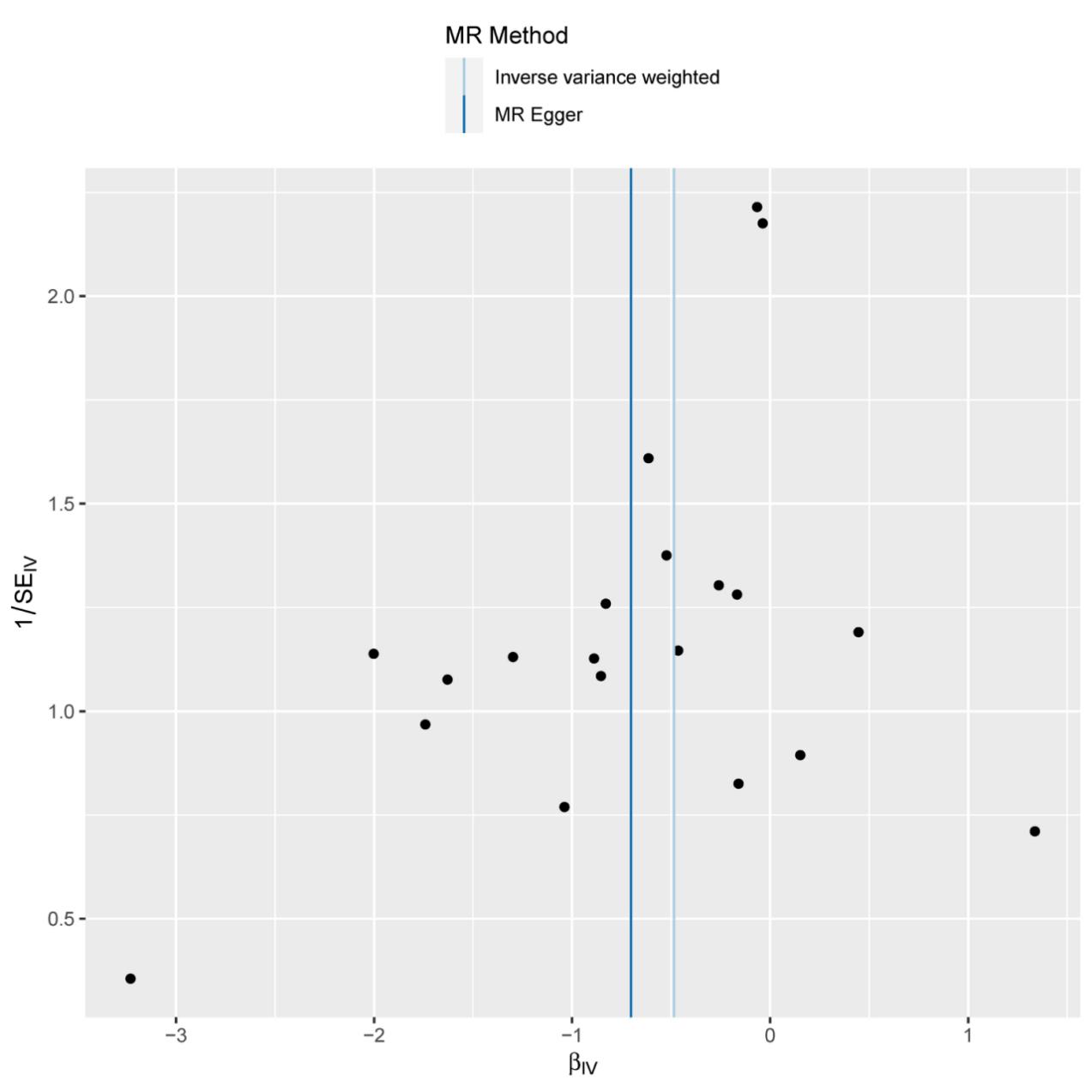


Funnel plot of genetic association estimates for Aspartate to citrate ratio
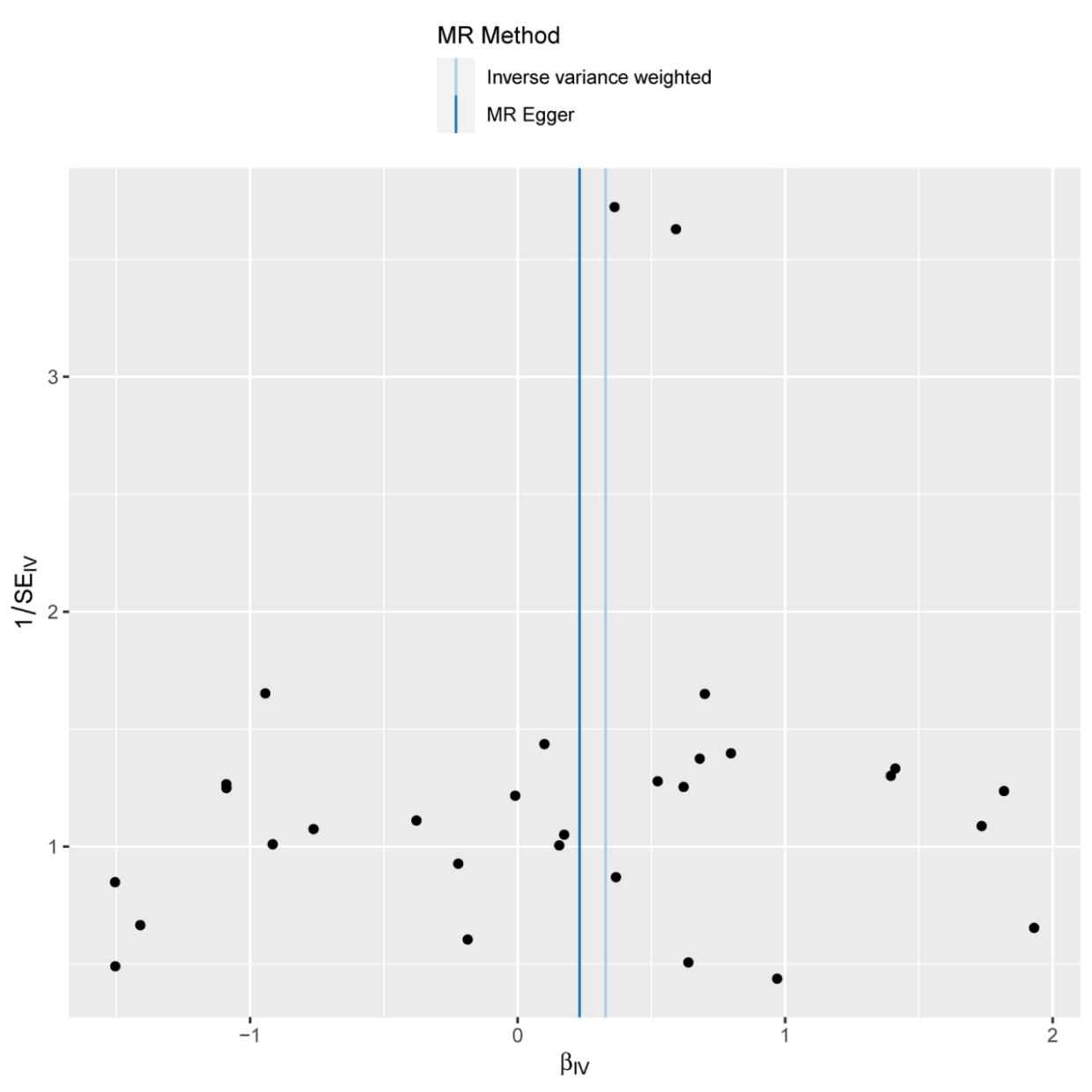


Funnel plot of genetic association estimates for Phosphate to mannose ratio


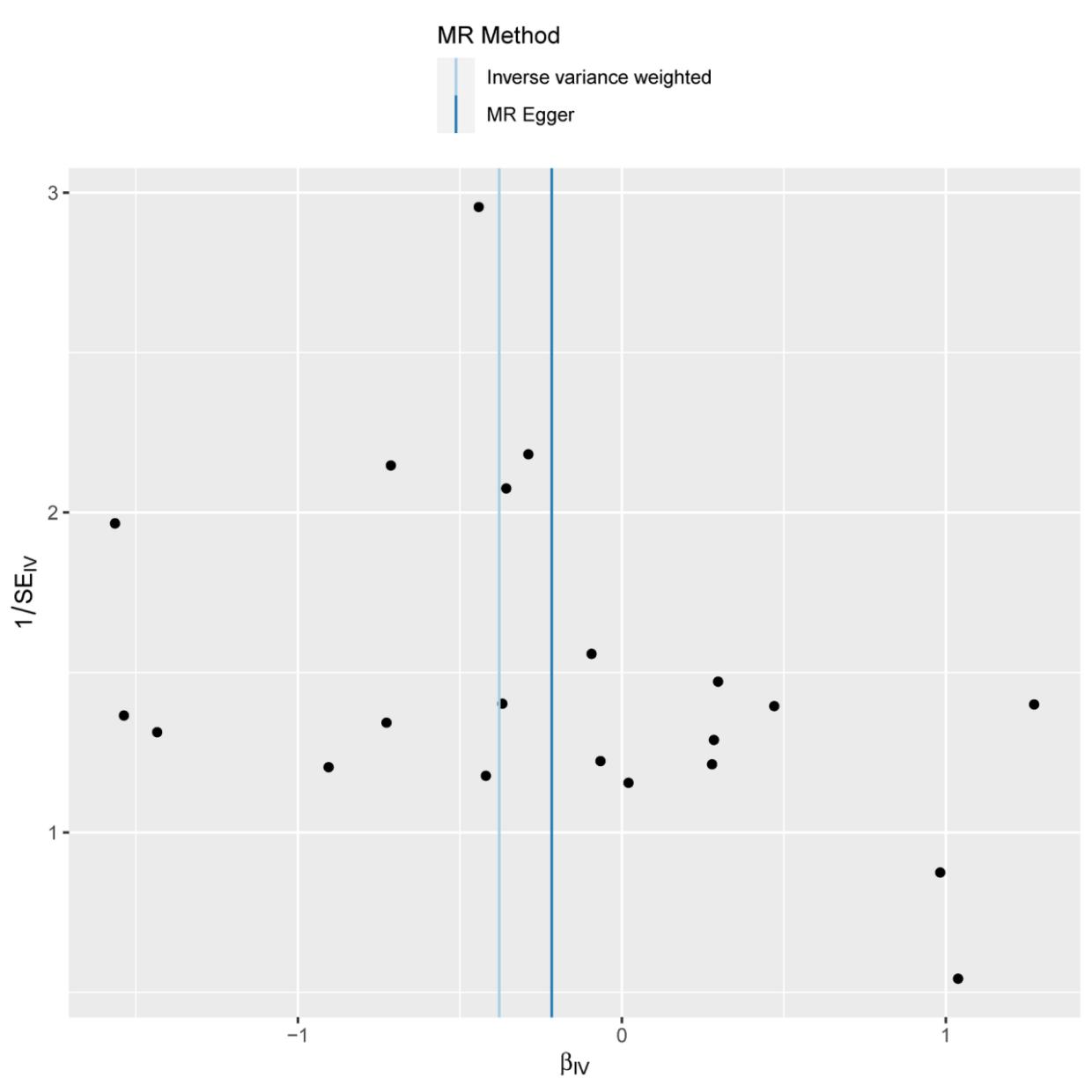


Funnel plot of genetic association estimates for Spermidine to ergothioneine ratio


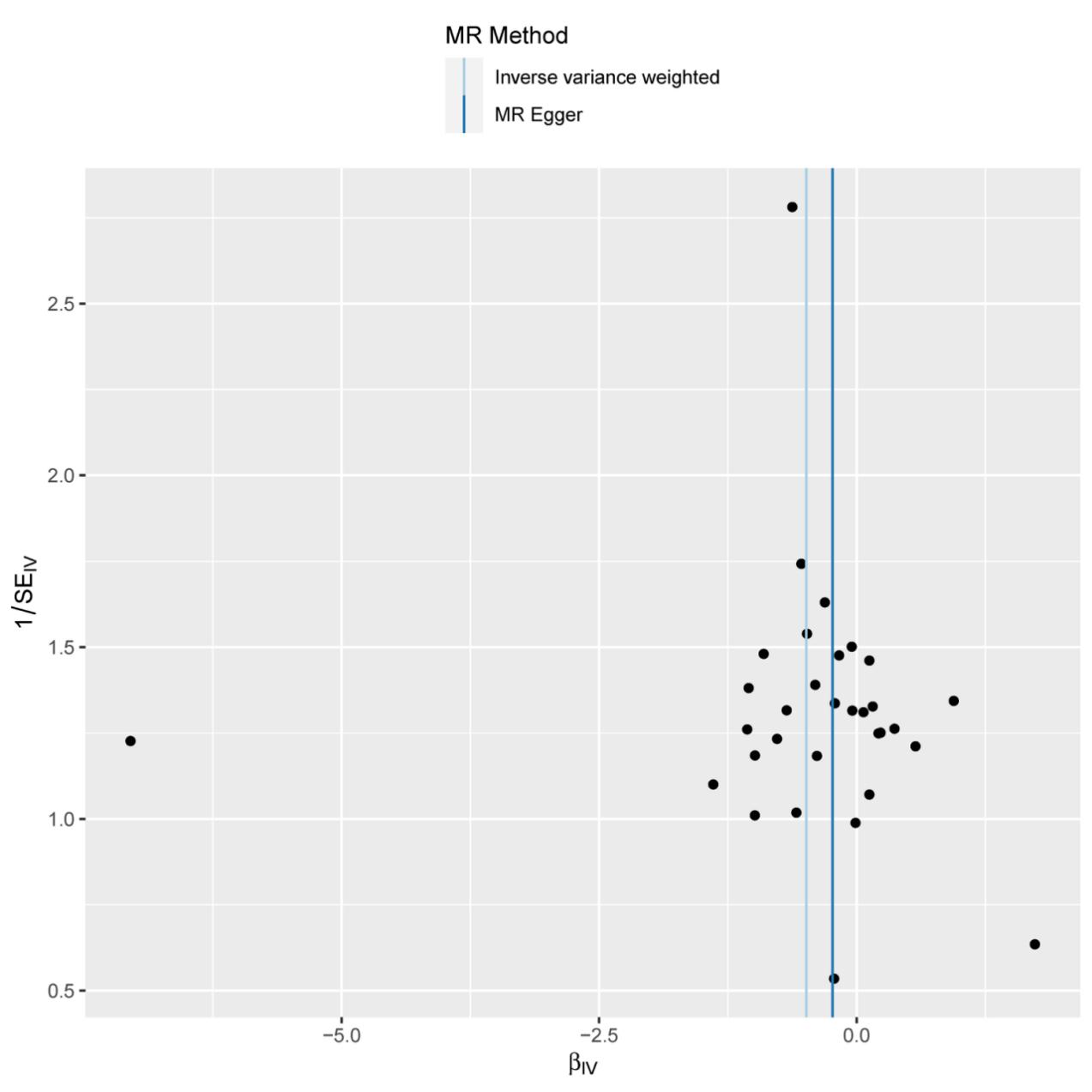


Funnel plot of genetic association estimates for Uridine to 2'-deoxyuridine ratio
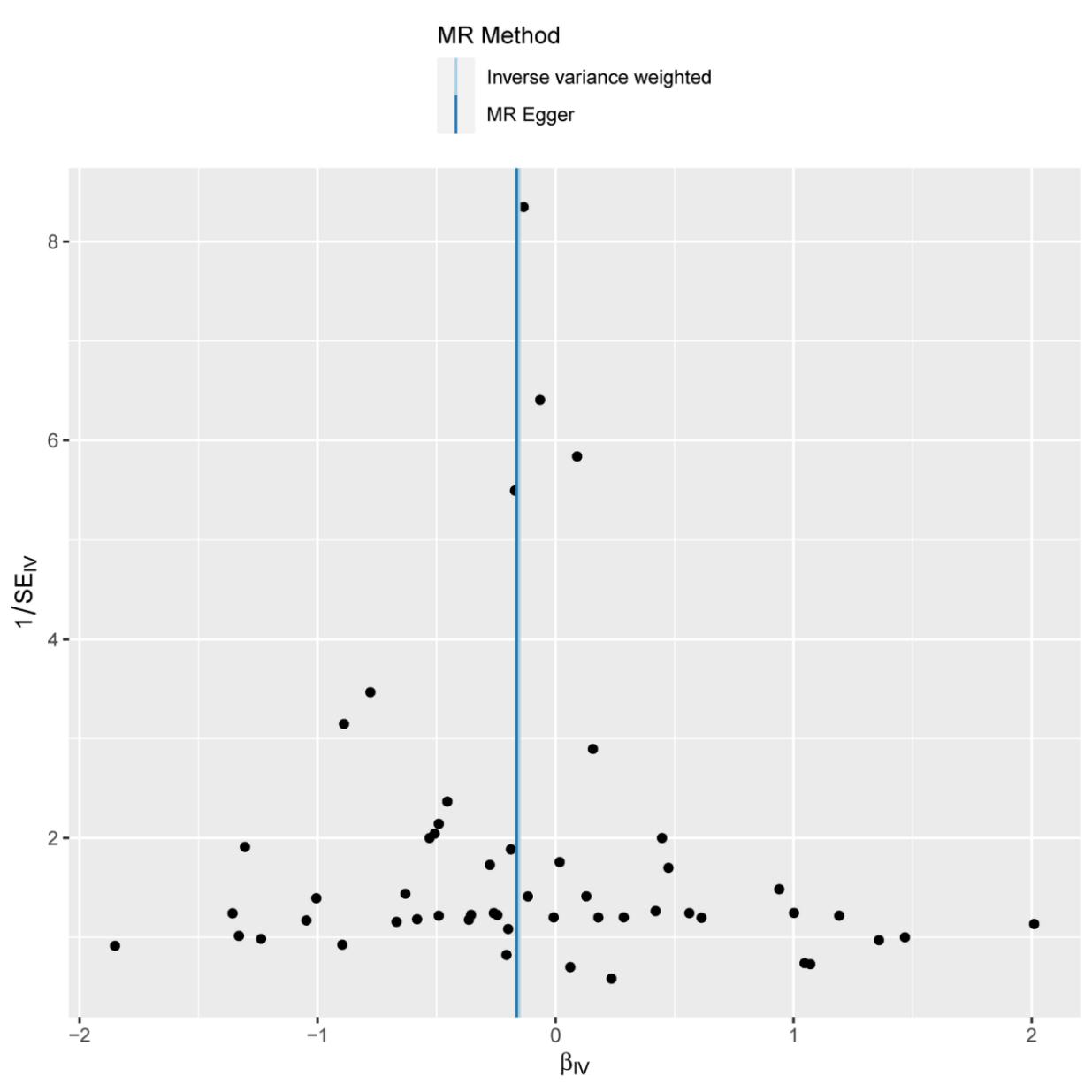


Funnel plot of genetic association estimates for Metabolonic lactone sulfate levels
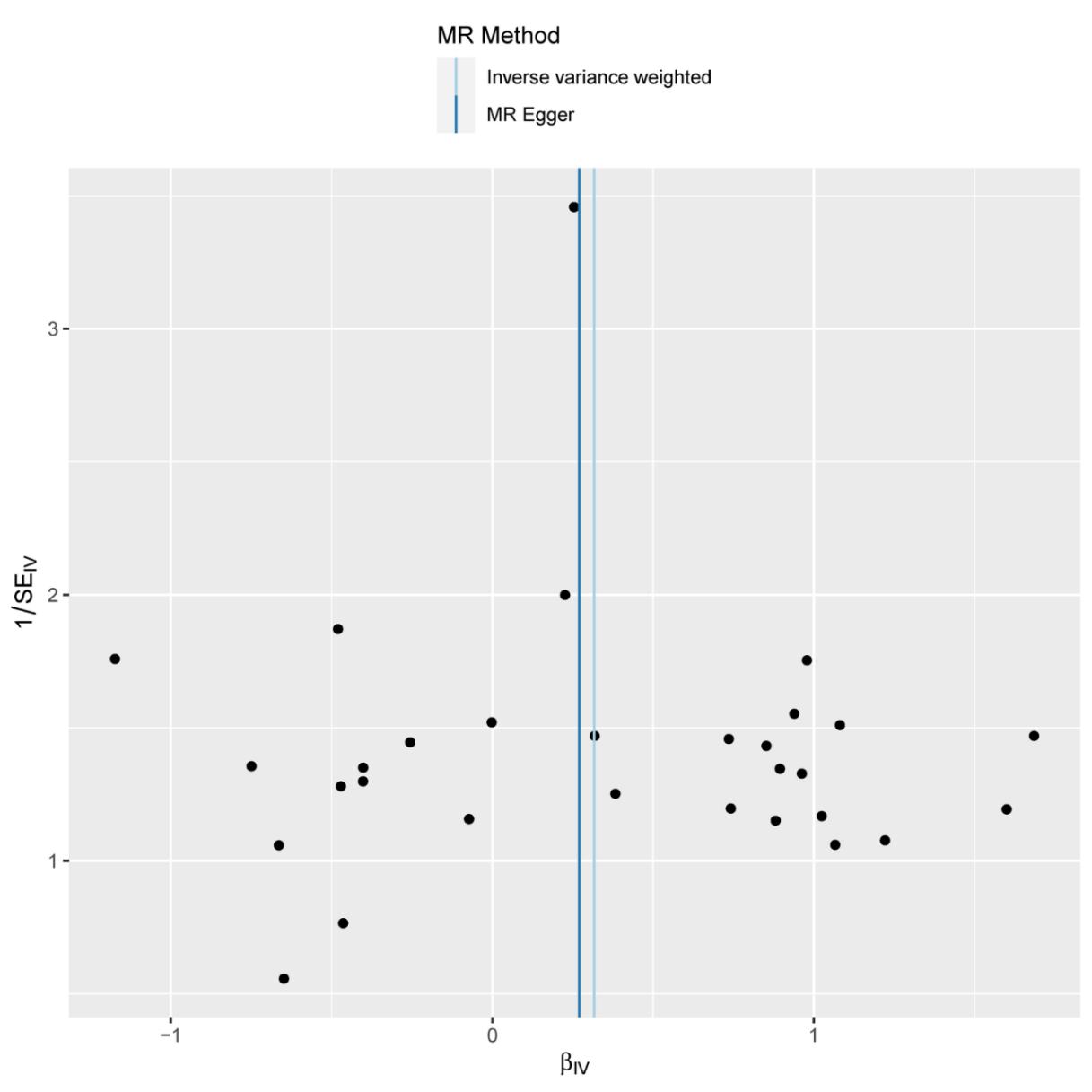


Funnel plot of genetic association estimates for X-12283 levels
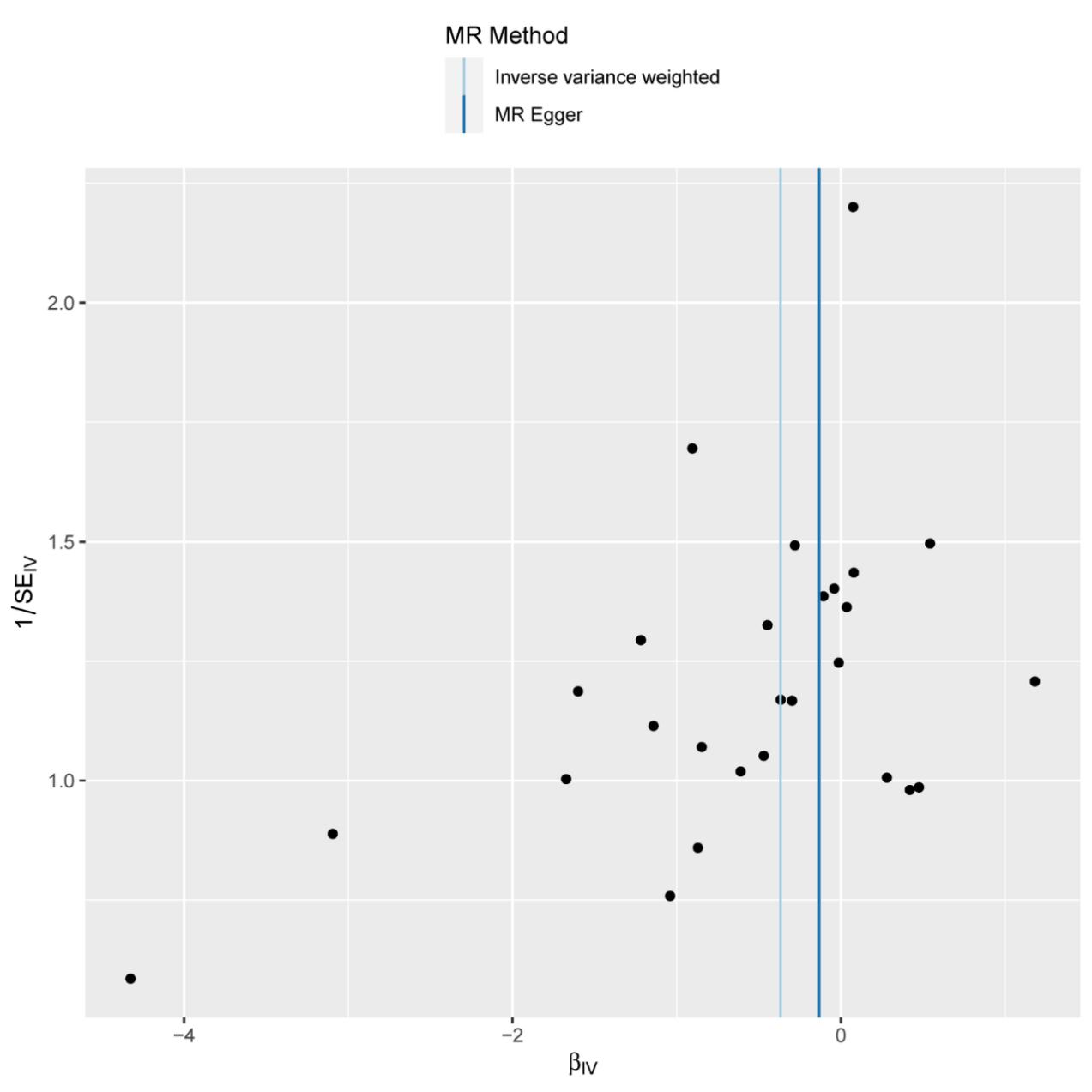


Funnel plot of genetic association estimates for X-13866 levels


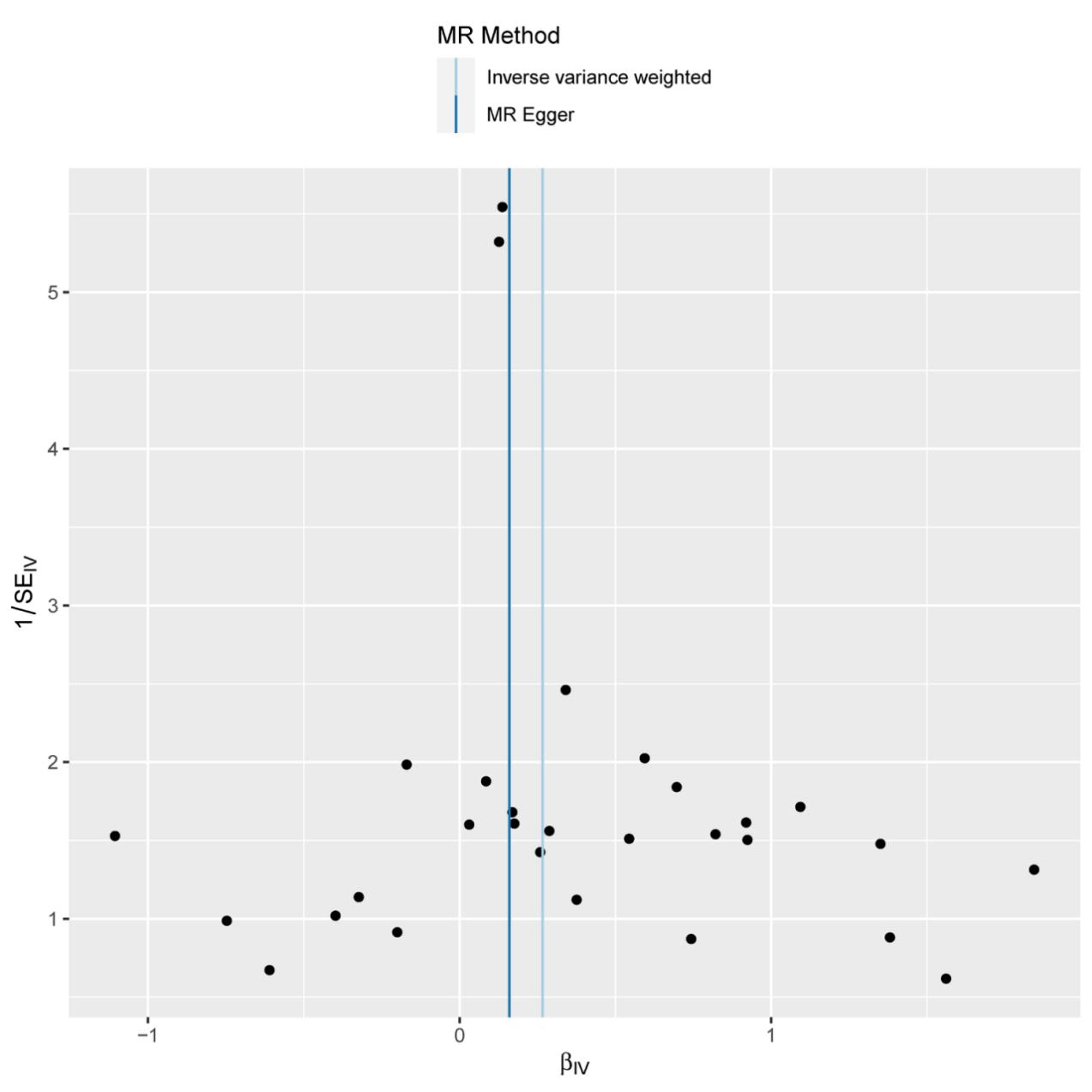


Funnel plot of genetic association estimates for X-16124 levels


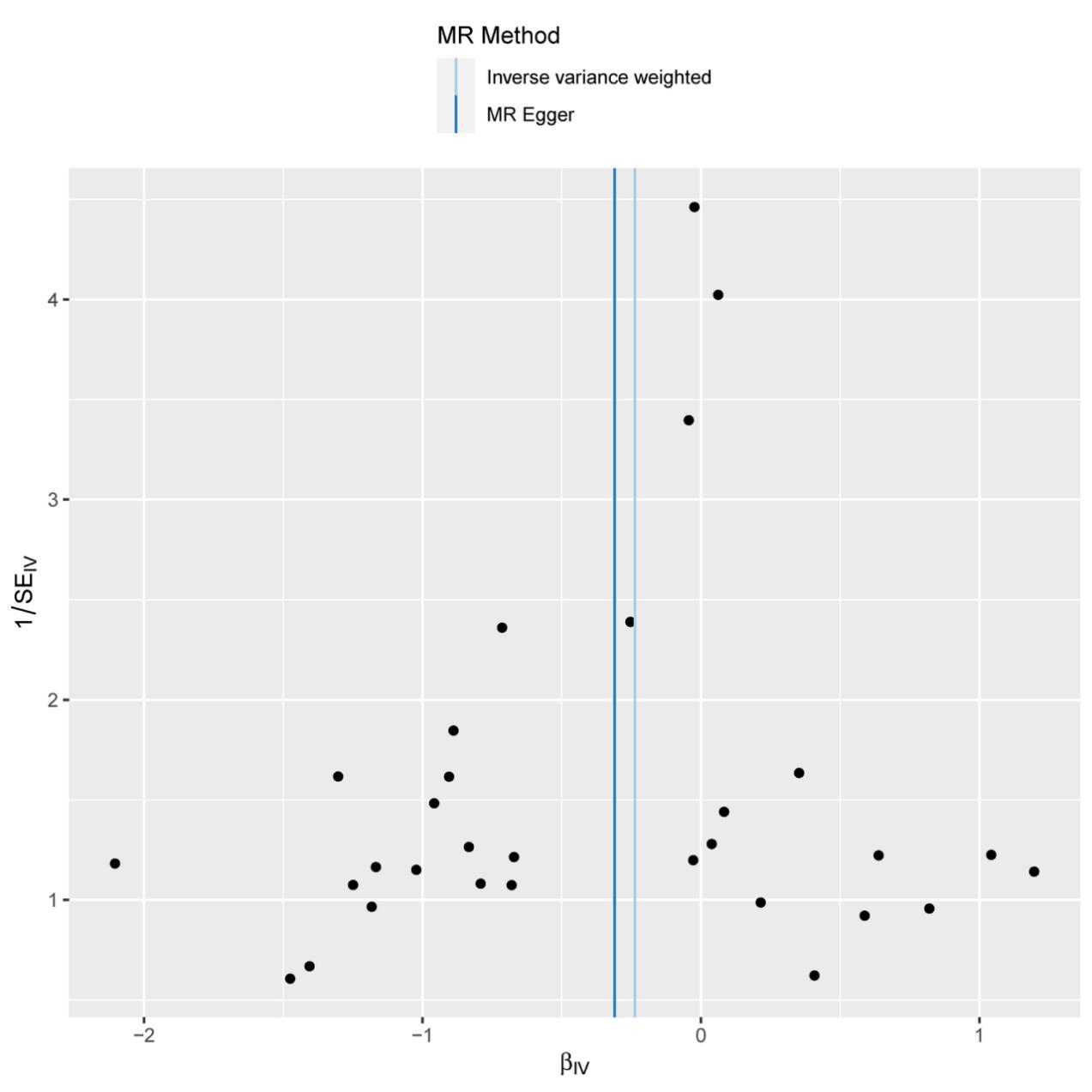


Funnel plot of genetic association estimates for X-21607 levels


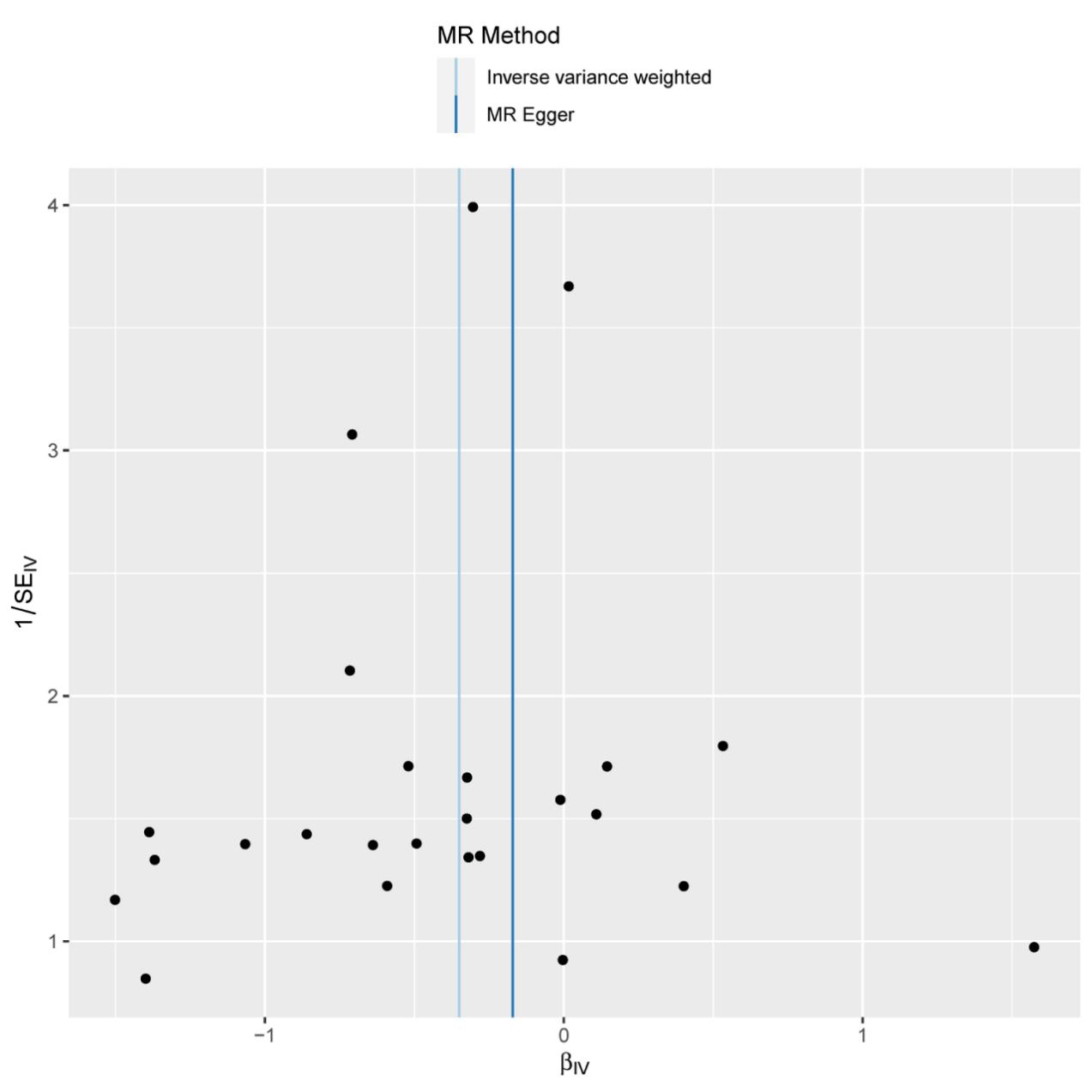


Funnel plot of genetic association estimates for X-24949 levels


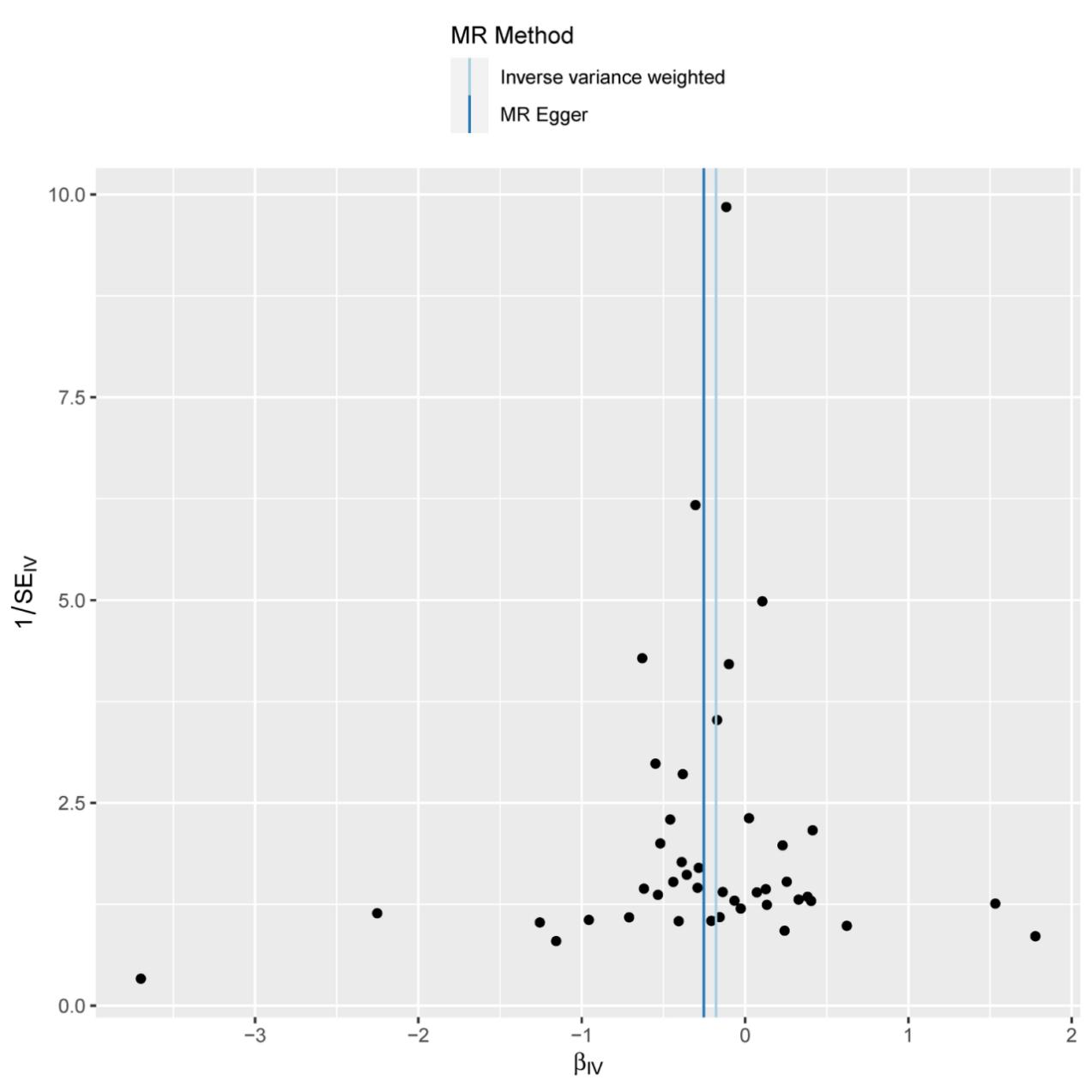


Funnel plot of genetic association estimates for X-26109 levels
